# Sensitive detection of minimal residual disease and immunotherapy targets by multi-modal bone marrow analysis in high-risk neuroblastoma – a multi-center study

**DOI:** 10.1186/s13046-025-03481-w

**Published:** 2025-08-02

**Authors:** Nina U. Gelineau, Eva Bozsaky, Lieke M. J. van Zogchel, Fikret Rifatbegovic, Daria Lazic, Andrea Ziegler, Ahmad Javadi, Lily Zappeij-Kannegieter, Ulrike Pötschger, Marta Fiocco, Peter F. Ambros, Inge M. Ambros, Bernd Bodenmiller, Ellen C. van der Schoot, Ruth Ladenstein, Marie Bernkopf, Godelieve A. M. Tytgat, Sabine Taschner-Mandl

**Affiliations:** 1 Princess Máxima Center for Pediatric Oncology, Research, Utrecht, The Netherlands; 2 Sanquin Research and Landsteiner Laboratoryof the AMC‐ University of, Department of Experimental Immunohematology, Amsterdam, the Netherlands; 3 St. Anna Children’s Cancer Research Institute, Vienna, Austria; 4 Mathematical Institute, Leiden University, Leiden, the Netherlands; 5 Department of Biomedical Data Science, Section Medical Statistics Section, Leiden University Medical Center, Leiden, The Netherlands; 6 Labdia Labordiagnostik GmbH, Vienna, Austria; 7 Institute of Molecular Health Sciences, ETH Zurich, Zurich, Switzerland; 8 Division Genetics, University Medical Center Utrecht, Utrecht, the Netherlands

**Keywords:** Neuroblastoma, Liquid biopsies, Minimal residual disease, Bone marrow, RT-qPCR, Automated immunofluorescence, Metastasis

## Abstract

**Background:**

Bone marrow dissemination of tumor cells, common in various cancers, including neuroblastoma, is associated with poor outcome, necessitating sensitive detection methods for bone marrow minimal residual disease (MRD) and offer detection of biomarkers for therapy stratification. Current standard-of-care diagnostics, involving cytomorphological and histological assessment of bone marrow aspirates and trephine biopsies, lack sensitivity, leading to undetected MRD in many patients, and do not allow molecular biomarker assessment.

**Methods:**

This study evaluates advanced multi-modal high-sensitivity MRD detection techniques in 509 bone marrow specimens from 108 high-risk neuroblastoma patients across two centers. We employed automatic immunofluorescence plus interphase fluorescence in situ hybridization (AIPF) and reverse transcriptase quantitative polymerase chain reaction (RT-qPCR) panels to quantify disseminated tumor cells (DTCs), disialoganglioside 2 (GD2) and CD56/Neural cell adhesion molecule (NCAM) levels, and adrenergic (ADRN) and mesenchymal (MES)-phenotype mRNA markers.

**Results:**

This multi-modal analysis significantly improved MRD detection compared to standard-of-care methods; 395 samples yielded results for RT-qPCR-ADRN, AIPF and CM/histology and 223 showed concordant results (64 positive, 159 negative). 114 samples did not produce results as either no cytospins were prepared (*n* = 96) or results were inconclusive (all techniques *n* = 18). AIPF and RT-qPCR complemented each other in detecting MRD and characterizing ADRN- and MES-phenotypes and GD2 immunotherapy target. RT-qPCR-ADRN alone frequently detected low tumor cell burden. High DTC infiltration at diagnosis showed bilateral bone marrow disease, whereas MRD settings often involved only one side. RT-qPCR-MES, despite lower sensitivity, identified 37 additional cases and showed delayed clearance of MES markers post-chemotherapy, with increases prior to relapse.

**Conclusions:**

Our findings demonstrate the feasibility of integrating high-sensitivity techniques with standard-of-care assessments in an international multicenter setting. Advanced multi-modal MRD detection, monitoring phenotype switches and assessing immunotherapy targets are crucial for improving patient outcomes in neuroblastoma and other cancers.

**Supplementary Information:**

The online version contains supplementary material available at 10.1186/s13046-025-03481-w.

## Statement of translational relevance

The findings from this study have significant implications for the future practice of neuroblastoma management. By showing the feasibility and effectiveness of using multi-modal, high-sensitivity minimal residual disease (MRD) detection techniques, i.e. automatic immunofluorescence plus interphase fluorescence *in situ* hybridization (AIPF) and reverse transcriptase quantitative polymerase chain reaction (RT-qPCR), in an international multicenter setting, this research paves the way for more standardized and accurate MRD assessments. These methods allow for early detection of disease progression and relapse, enabling timely intervention and personalized treatment adjustments. Additionally, the ability to monitor the plasticity of tumor cell states and the expression of disialoganglioside 2 (GD2) immunotherapy targets provides critical insights into therapy resistance and potential relapse. This approach not only enhances the precision of current MRD detection but also supports the optimization of immunotherapy by identifying appropriate targets on rare, therapy-resistant cells (in 5/108 patients). Overall, these advances in MRD detection and monitoring could significantly improve patient outcomes by facilitating tailored treatment strategies and earlier interventions. It will be important to assess the impact on patient survival prospectively in future studies.

## Background

Liquid biopsies are minimally invasive and allow for frequent and less burdensome disease diagnosis, prognosis and monitoring of cancer [1, 2]. A variety of liquids and analytes have demonstrated benefits in preclinical and clinical studies, e.g. tumor-specific mRNA, circulating or disseminated tumor cells, therapeutic targets or circulating cell-free DNA in bone marrow, blood, urine and other body fluids [3–5]. Bone marrow dissemination is frequently found in adult and pediatric cancers, such as breast cancer, prostate cancer, sarcomas and neuroblastoma, and is associated with poor prognosis in patients [6–8]. Therefore, monitoring disease in bone marrow liquid biopsies is critical for assessing therapeutic targets, therapy response and imminent disease recurrence.

Neuroblastoma is the most common extra-cranial solid tumor in children and patient outcomes largely depend on risk-group allocation according to international guidelines [9, 10]. High-risk patients are characterized by widely metastatic disease, including bone marrow involvement, genetic alterations (such as *MYCN* oncogene amplification), older age at initial diagnosis and other clinical parameters [9, 10]. Despite intensive multi-modal treatment in international study groups, less than 50% of patients survive [10–12]. At diagnosis, more than 90% of high-risk patients present with bone marrow infiltration [13, 14] and the majority of recurrent disease originates in the bone marrow, often deriving from a clone that was already present at time of diagnosis [15–17]. The current standard-of-care for assessing bone marrow disease advises to obtain two bone marrow aspirates and two trephine biopsies, one each from the right and left iliac crest [18, 19]. Bone trephine biopsies are histologically analyzed for tumor cells and bone marrow aspirates are assessed by cytomorphological staining of smears followed by visual inspection, which yields limited sensitivity and specificity. Yet, around 60% of patients assumed to be in complete remission by standard-of-care diagnostics experience recurrent disease, indicating that minimal residual disease (MRD) remains undetected [11]. Thus, there is a need for more sensitive and specific techniques to detect MRD in bone marrow that are also less labor-intensive and easy to standardize.

The effective quantification of tumor cells and therapeutic targets in the bone marrow is further challenged by the plasticity of neuroblastoma cells, which can assume adrenergic (ADRN) and mesenchymal (MES) cell states [20–22]. These cell states are characterized by distinct gene expression profiles and the activity of gene regulatory networks, such as (high) expression of paired-like homeobox 2b (*PHOX2B*), tyrosine hydroxylase (*TH*), cholinergic receptor nicotinic alpha 3 (*CHRNA3*) and growth-associated protein 43 (*GAP43*) in ADRN cells, and periostin (*POSTN*) and paired related homeobox 1 (*PRRX1*) in MES tumor cells [20, 23, 24]. MES neuroblastoma cells have been implicated in therapy resistance, at least in vitro and MES-related gene expression signatures are enriched in tumors during induction chemotherapy and in patients who experience relapse [20]. In vitro, the MES cell state also presents with low or absent levels of disialoganglioside 2 (GD2) and CD56 (neural cell adhesion molecule (NCAM)), both targets for immunotherapies [25], likely leading to therapy escape. Anti-GD2 antibody therapy has been the standard-of-care maintenance therapy for patients with high-risk neuroblastoma for the past decade and has led to a significant increase in event-free and overall survival [26]. However, anti-GD2 antibody therapy has been administered without stratifying for GD2 levels, which might explain the limited response in some patients [26]. Therefore, the assessment of MES cell states and the abundance of immunotherapy targets might be essential for accurately evaluating immunotherapy efficacy. While multiparameter flow cytometry is a sensitive method for assessing CD56 and GD2 expression and quantification for treatment purpose, techniques that enable more precise evaluation of tumor cell states and therapeutic targets are needed [27–29].

We have previously developed highly sensitive and specific assays to quantify (1) disseminated tumor cells (DTCs) in bone marrow aspirates, (2) GD2 and CD56 (NCAM) cellular levels and (3) ADRN- and MES-type tumor cells based on expression of specific mRNAs [24]. Automatic immunofluorescence plus interphase fluorescence in situ hybridization (iFISH) (AIPF), developed by Méhes et al [30]., allows the exact quantification of DTCs in the bone marrow using an automated microscopy imaging platform and unambiguous genetic verification. This technique sensitively detects up to one DTC in one million healthy bone marrow cells of neuroblastoma patients [30]. Another technique that can very sensitively detect tumor-derived mRNA in the bone marrow of patients with neuroblastoma is reverse transcriptase quantitative polymerase chain reaction (RT-qPCR) [19, 20, 31–40]. This technique reliably detects tumor cells with a sensitivity of one in one million normal nucleated bone marrow cells [41, 42]. We have previously designed two panels of neuroblastoma-mRNA markers for highly sensitive MRD monitoring in bone marrow: an ADRN- and a MES mRNA-panel [24].

In this prospective study across two centers, the main aim was to evaluate quantitative MRD in patients with high-risk neuroblastoma using AIPF and RT-qPCR against the standard-of-care, i.e. cytomorphological and histological assessment. Second, in the same sequentially collected bone marrow samples, we determined ADRN and MES cell states as well as GD2 and CD56 (NCAM) cellular expression. Furthermore, we evaluated and reported quality assessment and key performance parameters of these diagnostic tests, which will facilitate their rapid implementation as new standard-of-care.

## Methods

### Patients and samples

Bone marrow samples from high-risk neuroblastoma patients enrolled in the SIOPEN/HR-NBL1 (NCT01704716) [26, 43] or Dutch DCOG NB2009 (NCT01704716) [44] trials were prospectively collected at diagnosis and during treatment between 2018 and 2022 (Supplemental Fig. 1). The study was approved by the Medical Research Ethics Committees of the Academic Medical Center (Amsterdam, the Netherlands; MEC07/219#08.17.0836) and the Medical University of Vienna (Vienna, Austria; EK#1853/2016, EK#1216/2018), following the Declaration of Helsinki, with written informed consent obtained from parents/guardians [45]. Patients were diagnosed and staged per the International Neuroblastoma Staging System (INSS) [9, 46], with high-risk patient cases defined as stage 4 patients over one year old or any stage with *MYCN* amplification.

Bilateral bone marrow aspirates were obtained from left and right iliac crests and collected in ethylenediaminetetraacetic acid (EDTA) tubes. Within 24 h, cellular fractions were stored in Trizol (Invitrogen, Carlsbad, CA) at −80 °C (the Netherlands) or transferred to PAXgene blood RNA tubes (QIAGEN, Venlo, the Netherlands) and stored at −20 °C (Austria). RNA isolation and RT-qPCR were performed in Amsterdam, the Netherlands, while AIPF analysis took place in Vienna, Austria. Bone marrow histology and cytomorphology were centrally reviewed. Cytomorphological (CM) examination of Wright-Giemsa-stained smears was performed independently at St. Anna Children’s Hospital (Vienna, 10% cutoff) and the Princes Maxima Centre (Utrecht, the Netherlands, 1% cutoff), following respective study protocols. CM and histology were reported as either “positive”, “negative”, “inconclusive”, “dubious”, or “suspicious”. In this study, inconclusive and dubious samples were classified as negative, while suspicious samples were considered positive.

### mRNA extraction and RT-qPCR

mRNA extraction, complementary DNA (cDNA) synthesis and RT-qPCR followed established protocols [24]. mRNA was isolated from PAXgene blood RNA tubes (QIAGEN) using the PAXgene Blood RNA Kit (QIAGEN), or from cellular fractions stored in Trizol (Invitrogen), per manufacturer instructions. RNA concentration and quality were measured using an ND-1000 spectrophotometer (Nanodrop). cDNA synthesis was done using the High-Capacity RNA-to-cDNA™ Kit (Applied Biosystems, Foster City, CA, USA) [24]. RT-qPCR was performed on the Viia7 (Applied Biosystems, Carlsbad, CA, USA) and analyzed using QuantStudio software v1.6 (Applied Biosystems). The housekeeping gene glucuronidase beta (*GUSB*) and the neuroblastoma-specific mRNA marker *PHOX2B* were used as single markers [24]. ADRN markers (*TH, CHRNA3* and *GAP43*), and MES markers (*POSTN*, *PRRX1* and flavin-containing monooxygenase 3 (*FMO3*)), were performed in multiplex panels [24]. mRNA expression was normalized to *GUSB* (ΔCt = Ct_marker_ – Ct_*GUSB*_). Samples with both *GUSB* Ct > 25 and *PHOX2B* negativity were excluded. A sample was scored positive if at least one of the markers scored positive, according to previously published thresholds [47]. Level of mRNA detection (‘infiltration’) was calculated relative to neuroblastoma cell line IMR32 (2^ΔΔCT (ΔCT_sample_ – ΔCT_IMR32_) * 100%). Median relative expression of positive markers was used to calculate infiltration level. Due to the calculations made relative to the expression in IMR32, calculated infiltrations can exceed 100%. For bilateral samples, the highest infiltration level was used. MES markers *POSTN* and *PRRX1* were scored based on previously published thresholds relative to both *GUSB* and *FMO3* expression [47]. High expression of *POSTN* or *PRRX1* and low expression of *FMO3* identify MES neuroblastoma cells in bone marrow samples.

### Automatic immunofluorescence plus iFISH (AIPF) analysis

Bone marrow aspirates were collected in EDTA tubes and subjected to density gradient centrifugation to isolate the mononuclear fraction, which also contains disseminated neuroblastoma cells. Cytospins (0.8–1.5 × 10^6^ mononuclear cells (MNCs) per slide) were prepared, fixated and immunocytologically stained with labeled antibodies for GD2 and CD56 (Anti-GD2-FITC, Anti-CD56-Biotin, Mouse-Anti-Biotin-Cy3) (Supplemental Table 1A). Automated imaging (Axioplan 2, Zeiss, TRITC, FITC and DAPI filter) and analysis (Metafer 4, RCDetect classifier, Metasystems) identified DTCs [30, 48]. The total bone marrow MNC count per slide was extrapolated from representative measurements and GD2/CD56-positive cells were counted as DTCs. If fewer than four DTCs or ambiguous cells were detected, iFISH was performed using FISH probes (Metasystems) for primary tumor-specific aberrations (e.g., MYCN amplification, 1p deletion, 17q gain). Each analysis included positive and negative controls. If a sample was negative for both GD2 and CD56, no GD2- and/or CD56-positive tumor cells were detected. Infiltration was reported as the fraction of DTCs within MNC count [30, 48]. Detailed protocols are provided in Supplemental Data.

### Imaging mass cytometry

Archived bone marrow cytopins from patient 166 with metastatic neuroblastoma were analyzed using imaging mass cytometry for twelve tumor-specific markers (antibodies against GD2, CD56/NCAM, CHGA, S100B, CXCR4, ELAVL4, GATA3, PRPH, SOX10, Vimentin, CD44, CD24) (Supplemental Table 1B), markers for nucleus detection and Ki-67 as a proliferation marker, as described in Lazic et al. (personal communication). Patient 166 was chosen due to the availability of multiple sequential timepoints in our study. Samples were thawed for 15 min at RT, fixed in 4% PFA (Carl Roth) at 4 °C for 30 min, washed twice in TBS and then blocked with 2% BSA (Carl Roth) and 0.1% Tween-20 (Merck) in TBS at RT for 1 h. Meanwhile, antibody cocktails were retrieved from −80 °C and thawed for 15 min at 4 °C before staining overnight at 4 °C. The following day, slides were washed twice (5 min per wash) in TBS, stained with the DNA intercalator Iridium (Fluidigm) and then dried with compressed air before IMC measurement with the Hyperion imaging system (Fluidigm) and CyTOF Software v7. Acquired images were background-corrected using spillover compensation with R CATALYST v.1.20.1 package [49], hot pixel removal through IMC-Denoise [50] and semi-automated background correction [51]. Nuclei were segmented using a Cellpose model fine-tuned in a human-in-the-loop approach with Cellpose 2.0 [52]. Single-cell mean marker expressions were subsequently exported based on inferred segmentation masks.

### Statistical analysis

Statistical analysis was performed using SPSS v29 (RRID:SCR_002865; IBM Corporation, Armonk, NY, USA). Continuous variables were reported as median (10-90th percentiles), mean ± standard deviation, or mean (quartiles). Spearman correlations (95% confidence interval (CI)) assessed AIPF and RT-qPCR infiltration levels in paired samples. Independent samples t-test or Mann–Whitney U tests (for non-normal distributions) compared continuous variables. Graphs were generated using GraphPad Prism v9.3.1 (RRID:SCR_002798; GraphPad Software, Boston, Massachusetts, USA).

## Results

### Multi-modal bone marrow disease analysis is feasible in a multi-center setting

**Fig. 1:**
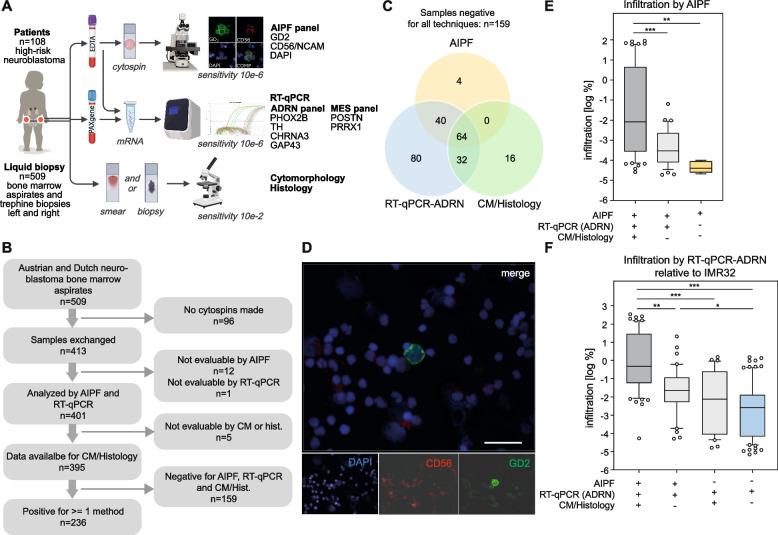
Study design, cohort and benchmarking against standard of care bone marrow assessment. **A** Outline of study cohort and analytical workflows for automated immunofluorescence plus fluorescence in situ hybridization (AIPF), RT-qPCR and cytomorphology/histology. **B** Consort diagram depicting sample inclusion and exclusion. **C** Contribution of AIPF, RT-qPCR (adrenergic (ADRN)-mRNA markers) and cytomorphology (CM)/histology. Venn diagram shows samples positive for at least one technology. Each circle represents positive results of one technique. *N* = 395 samples analyzed by all three techniques. *N* = 236 samples positive by ≥ 1 technique. **D** Representative microscopy image for GD2, CD56/NCAM and DAPI for a bone marrow specimen that was identified as positive only by AIPF, and negative by RT-qPCR and CM/histology. Scale bar represents 20 µm. **E** Level of tumor cell infiltration according to AIPF (y-axis; give n as % DTCs detected by AIPF) in specimens with single or combined positivity for AIPF, RT-qPCR and CM/histology (x-axis; + positive,—negative). Box plots represent 10–90 percentiles, line shows median. ** = 0.0023; *** = 0.0003. **F** Level of tumor cell infiltration according to RT-qPCR-ADRN (y-axis; given as % relative to neuroblastoma cell line IMR32) in specimen with single or combined positivity for AIPF, RT-qPCR-ADRN and CM/histology (x-axis; + positive,—negative). Box plots represent 10–90 percentiles, line shows median. * = 0.0135; ** = 0.0007; *** < 0.0001

One hundred eight (108) children with high-risk neuroblastoma, with a median age of 36 months at diagnosis (range 3–274 months) were included in this study (Table 1). Five hundred nine (509) bone marrow samples were collected (Fig. 1A & B), shipped and analyzed in specialized laboratories for AIPF and RT-qPCR. The current standard-of-care diagnostic tests, i.e. CM or histology, were performed locally. To investigate the feasibility of performing multi-modal bone marrow disease analysis on the same sample, we first evaluated how often each technique was successfully performed in our cohort (Table 2). Due to low cellularity in 96 samples (19%), no cytospins could be made and thus AIPF was performed on 413 samples. Twelve (3%) were not evaluable by AIPF due to high autofluorescence or low cell counts. Sequential iFISH analysis visualizing tumor-typical cytogenetic aberrations was performed in forty ambiguously positive samples. In 25/40 (63%), iFISH confirmed the same number of positive cells as with automated immunofluorescence alone. In 15/40 (38%), the number of positive cells detected by AIPF differed from that detected by automated immunofluorescence. However, only in 7/15 (47%), iFISH-result classified samples as negative or led to inconclusive results. Interestingly, 5 of these 7 (71%) were RT-qPCR positive. Of the 509 samples collected, neuroblastoma mRNA detection failed in nine (2%) because of low cDNA concentration. However, in eight of these cases, the paired bone marrow sample collected from the contralateral side yielded sufficient cDNA and could be included in the analyses (yielding 508). Standard-of-care diagnostics (CM or histology) was performed in 505 samples of which 1/505 (0.2%) was not evaluable by either, CM or histology (118 CM only, 2 histology only, 384 CM and histology) (Fig. 1B and Table 2).

**Table 1:** Patient characteristics

Number of patients	108
Country	
Austria	39
Netherlands	69
Stage (INSS)	
3	2
4	105
Unknown	1
Age at diagnosis (months)	
< 18	25
≥ 18	83
Median	36
Range	3–274
Sex	
Male	65
Female	43
*MYCN* amplification status	
Amplified	38
Non-amplified	69
Unknown	1

*INSS* International Neuroblastoma Staging System

**Table 2:** Technique performance on bone marrow liquid biopsy samples from patients with high-risk neuroblastoma

	**Samples analyzed** **[number]**	**Analysis successful** **[number]**		**Analysis failed ** **[number (%)]**		**Samples not analyzed [number (%)]**
AIPF*	413	401		12 (3%)		96 (19%)
**Cell numbers**			**iFISH** **performed**			
<1 million	32	29	1	3		
1-2 million	104	104	13	0		
≥2 million	264	264	25	0		
Unknown	13	4	1	9		
RT-qPCR*	509	508		1 (0.2%)	GUS Ct >25	Not applicable
					One side only: 8	
					Both sides: 1	
CM*	503	435	Suspicious: 2	68 (13.5%)	Not representative: 3	6
					Inconclusive: 63	
					Dubious: 2	
Histology*	387	333	Suspicious: 12	54 (14%)	Not representative: 5	122
					Inconclusive: 49	

^*^ Total number of samples collected *n* 509

Of the 413 samples exchanged, 184 (45%) were from Austrian and 229 (55%) from Dutch patients (Supplemental Fig. 1A). For > 95% of these, it was possible to perform AIPF and RT-qPCR, as well as cytomorphological examination of bone marrow smears and/or histological examination of bone trephines (Supplemental Fig. 1B). In total 395 samples were analyzed by AIPF, RT-qPCR and CM/histology (Fig. 1B). Sample preparation time (i.e. time from sampling to cytospin preparation), was shorter for Austrian bone marrow aspirates (Supplemental Fig. 1C). Mean cell counts were 3.80 million (range 130,000–7.97 million) for Austrian and 1.95 million (237,000–5.00 million) for Dutch samples (Supplemental Fig. 1D). The median turnaround time (sample collection to clinical report) for AIPF was 6 days (range 1 to 29 days), for the Austrian samples, where the results are used clinically. Two hundred thirty-six (236) bone marrows (60%) were positive for one or more techniques (Fig. 1B). We conclude that performing all three analyses on the same bone marrow specimen is feasible in an international multi-center setting.

### Multi-modal analysis is superior in detecting bone marrow disease as compared to single modalities

In the cohort of 395 multi-modal bone marrow analyses, we next determined the contribution of each technique to MRD detection and the link of DTC infiltration to uni- versus multi-modal assessment. In the 395 samples analyzed by all three techniques—AIPF, RT-qPCR-ADRN and CM/histology—64 (16%) were positive by all techniques and 159 (40%) negative. Among the 236 bone marrow specimens (60%) that showed positivity for at least one of the techniques, RT-qPCR-ADRN detected bone marrow disease more frequently than AIPF or CM/histology (216 RT-qPCR-ADRN^pos^ vs. 108 AIPF^pos^ vs. 112 CM/histology^pos^). Additionally, RT-qPCR-ADRN showed exclusive positivity in eighty, AIPF in four and CM/histology in sixteen cases (Fig. 1C & D & Supplemental Table 2). Interestingly, in CM/histology^neg^ samples (*n* = 283), MRD-detection by AIPF was positive in 16% (*n* = 44) and RT-qPCR-ADRN was positive in 42% (*n* = 120); two of these cases were taken at diagnosis: both were RT-qPCR^pos^ and one AIPF^pos^.

Thus, when comparing AIPF or RT-qPCR-ADRN against the standard-of-care, CM/histology, RT-qPCR-ADRN detected a significantly higher number of patients (*p* < 0.0001, Mc Nemar’s test), whereas AIPF did not (Table 3). The combination AIPF/RT-qPCR-ADRN identified 220/395 positive samples, 124 of which were negative according to CM/histology. A total of 112 were CM/histology^pos^, and sixteen of these were AIPF/RT-qPCR-ADRN^neg^. Accordingly, the combination detected a significantly higher number of patients (*p* < 0.0001, Mc Nemar’s test) (Table 3). When considering discordant pairs the number of samples that tested AIPF/RT-qPCR-ADRN^pos^ CM/histology^neg^ was 7.8-times higher as the number of samples that tested positive with CM/histology alone (95% CI, 4.6–13.0) (Table 3). The combination also outperformed RT-qPCR-ADRN alone (7.5-times; 95% CI, 4.5–12.6) and AIPF alone (0.9-times; 95% CI 0.6–1.4) (Table 3).

**Table 3:** Comparison of single assay performance versus combined assays against cytomorphology and histology (current standard-of-care)

		CM & Histology			Mc Nemar’s test		Discordant pairs
		Negative	Positive	Total	Chi-Square	*p*-value	Rel. frequency (range)
AIPF	Negative	239	48	287	0.2	0.7	0.9 (0.6-1.4)
	Positive	44	64	108			
RT-qPCR-ADRN	Negative	163	16	179	79.5	<0.0001	7.5 (4.5-12.6)
	Positive	120	96	216			
RT-qPCR-MES	Negative	201	87	288	0.1	0.7	0.9 (0.7-1.3)
	Positive	82	25	107			
Combined AIPF & RT-qPCR-ADRN	Negative	159	16	175	83.3	<0.0001	7.8 (4.6–13.0)
	Positive	124	96	220			
Combined AIPF & RT-qPCR-ARDN & RT-qPCR-MES	Negative	122	15	137	121.1	<0.0001	10.7 (6.3-18.2)
	Positive	161	97	258			

Infiltration of DTCs ranged from 1 DTC in 5.30 million bone marrow MNCs to 2.23 million DTCs in 2.48 million MNCs (90% tumor cell content) assessed by AIPF and 0.000007–341% infiltration relative to IMR32 for RT-qPCR-ARDN (Fig. 1E & F). In samples with positive results in all three techniques, infiltration (measured by AIPF and RT-qPCR-ADRN) was higher compared to samples positive by only AIPF or RT-qPCR-ADRN, or both. Disaggregation of data by country showed the same trend in sensitivity and a comparable fraction of positive samples (Supplemental Fig. 1E & F).

Together, these data demonstrate that multi-modal analysis is more effective at detecting bone marrow disease than the current standard of care or each methodology individually. Furthermore, low tumor cell burden is more frequently detected by RT-qPCR-ADRN alone and, less frequently, by AIPF.

### High tumor cell infiltration at diagnosis is associated with bilateral bone marrow involvement and shifts towards unilateral upon therapy-induced reduction of tumor cells

**Fig. 2:**
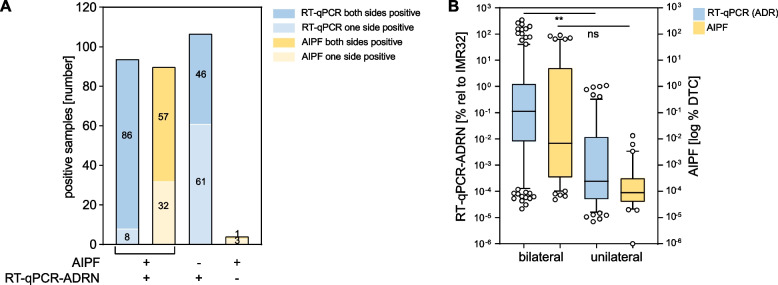
High tumor cell infiltration at diagnosis is associated with bilateral bone marrow involvement and shift towards unilateral upon therapy-induced reduction of tumor cells. **A** AIPF and RT-qPCR positivity on one side only (unilateral) and on both sides (bilateral) in samples where both sides were analyzed for AIPF and RT-qPCR-ADRN (*n* = 93 AIPF samples positive; *n* = 201 RT-qPCR-ADRN samples positive). **B** Level of tumor cell infiltration by RT-qPCR-ADRN (left y-axis) and AIPF (right y-axis) according to bilateral (RT-qPCR-ADRN *n* = 132; AIPF *n* = 53) and unilateral positive (RT-qPCR-ADRN *n* = 69; AIPF *n* = 36) result at all timepoints. ** = 0.0045; ns = not significant

Since bone marrow aspirates were obtained from two sites (left and right iliac crests), we compared results from paired bone marrow samples. For the bilaterally tested pairs, 176 AIPF pairs and 175 RT-qPCR-ADRN pairs had identical results for both sides: MRD-negative in 156 AIPF pairs and 121 RT-qPCR-ADRN pairs and MRD-positive in twenty AIPF pairs and 54 RT-qPCR pairs. For samples that were positive for both AIPF and RT-qPCR, the majority were positive on both sides (AIPF 57/89, RT-qPCR-ADRN 86/94) (Fig. 2A). In contrast, samples that were positive by one technique only, unilateral tumor cell infiltration was more often observed (RT-qPCR-ADRN 61/107, AIPF 3/4) (Fig. 2A). The DTC infiltration levels of bilateral positive specimens were significantly higher than the unilateral positive ones, reflecting low infiltration burden in the latter (Fig. 2B). However, there was no significant difference between the left and right sides (Supplemental Fig. 2A).

When the standard-of-care MRD diagnostics by CM and histology were included in the analysis, about half of samples positive for all techniques were bilaterally positive by CM. Whereas those only positive for RT-qPCR-ADRN or CM showed a higher frequency of unilaterally positive cases (Supplemental Fig. 2B). Interestingly, for two that were one-side-only positive, AIPF and RT-qPCR-ADRN were positive on opposite sides, while CM was negative in these cases.

Comparing different timepoints during the treatment course, the number of bilateral-positive samples decreased from diagnosis, when bilateral positivity was predominant (AIPF bilateral 26/37 (70%); RT-qPCR-ADRN 37/46 (80%)), versus post-induction chemotherapy (AIPF 9/19 (47%); RT-qPCR-ARDN 26/37 (70%)) and end of treatment, when only unilateral MRD was detected (RT-qPCR-ADRN bilateral 0/7) (Supplemental Fig. 2C). This shows that bone marrow disease is present at both sides when DTC infiltration is high, i.e. at diagnosis, while in MRD settings, during and end of treatment, more frequently only one side is affected.

### Automated microscopy AIPF and adrenergic mRNA markers are complementary in quantifying MRD and heterogeneity in immunotherapy targets

**Fig. 3:**
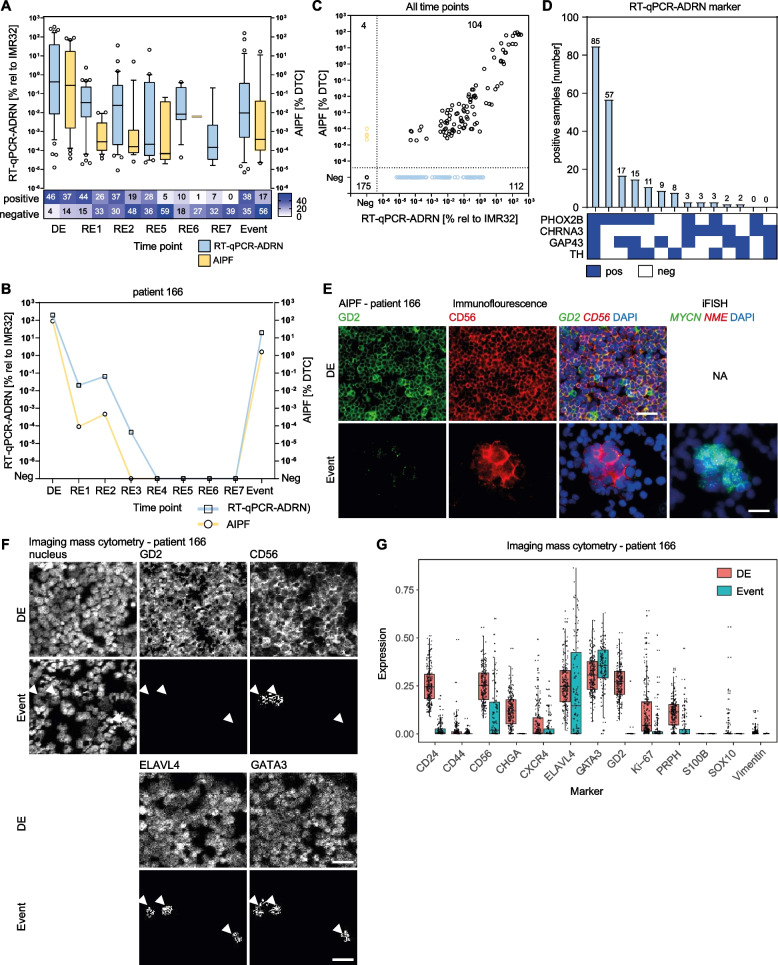
Adrenergic RT-qPCR is more sensitive to detect MRD and AIPF detects heterogeneity in immunotherapy targets GD2 and CD56/NCAM. **A** Level of tumor cell infiltration according to RT-qPCR-ADRN (left y-axis; given as % relative neuroblastoma cell line IMR32) and AIPF (right y-axis; given as % DTCs detected by AIPF) per timepoint during therapy and progression or relapse (event). Box plots represent 10–90 percentiles, line shows median. Heatmap represents number of negative and positive cases. **B** Representative case (patient 166) showing initial response to therapy and relapse. Bone marrow liquid biopsy assessed by RT-qPCR-ADRN (left y-axis; given as % relative to neuroblastoma cell line IM32) and AIPF (right y-axis; given as % DTCs) per timepoint. DE = diagnosis, RE1 = mid-induction chemotherapy, RE2 = end of induction therapy, RE3 = surgery, RE4 = before stem cell transplantation, RE5 = before immunotherapy, RES6 = mid-immunotherapy, RE7 = at end of immunotherapy. **C** Scatter plot showing level of infiltration according to AIPF (y-axis; given as % DTCs) and RT-qPCR-ADRN (x-axis; given as % relative to neuroblastoma cell line IMR32) in bone marrow from all timepoints analyzed by both techniques (*n* = 395; Spearman correlation = 0.69, 95% CI 0.57–0.78; *p* < 0.001 of samples positive for both techniques (*n* = 104)). **D** Adrenergic mRNA-marker (co-)expression by RT-qPCR on samples with positive result all timepoints (*n* = 215). **E** Automated immunofluorescence plus iFISH (AIPF) of patient 166 showing heterogeneous levels of GD2 and CD56/NCAM at event versus homogeneous GD2 at diagnosis (DE). Tumor cell identity was confirmed by iFISH for *MYCN* amplification and *NME* as reference. **F** Representative images of tumor marker protein expression by imaging mass cytometry (IMC) in patient 166 illustrating loss of GD2 at event (relapse). **G** Imaging mass cytometry (IMC) of patient 166. Tumor marker protein expression (y-axis) in tumor cells at diagnosis (DE) and event (relapse)

Given that multi-modal analysis was more sensitive to detect MRD, we investigated how levels of ADRN mRNAs and immunotherapy targets GD2 and CD56 correlated at different timepoints during high-risk treatment. Since AIPF can detect absolute neuroblastoma tumor cell counts and RT-qPCR detects neuroblastoma transcripts, we compared the level of calculated infiltration by AIPF with that calculated by RT-qPCR in paired samples. As expected, at diagnosis, ADRN mRNAs and the fraction of GD2^pos^CD56^pos^ cells were high and there was a strong correlation between the bone marrow infiltration of samples positive for AIPF and RT-qPCR (*n* = 37, Spearman correlation equal to 0.75, 95% CI 0.55–0.87; *p* < 0.001) (Fig. 3A & Supplemental Fig. 3A). During treatment, more bone marrow samples became negative and in those retaining tumor cells, the level of infiltration was reduced. At relapse, however, bone marrow disease was again detected by RT-qPCR-ADRN and AIPF (Fig. 3A). This kinetics of molecular detection of initial treatment response and subsequent relapse is exemplified by patient 166 (Fig. 3B). Correlation of infiltration levels was also high when samples during treatment or relapse were considered (*n* = 395; Spearman correlation 0.69, 95% CI 0.57–0.78; *p* < 0.001 of samples positive for both techniques (*n* = 104)) (Fig. 3C). When investigating the mRNA markers contributing to MRD detection, most samples were positive for all four ADRN markers: *PHOX2B, TH, CHRNA3* and *GAP43.* Although *PHOX2B* had the lowest median expression of all four investigated ADRN markers, it contributed the most as single marker (57/216; 26%) and in combinations (191/216; 88%), while only nine samples were *TH* or *GAP43* single positive and three for *CHRNA3* alone (Fig. 3D and Supplemental Fig. 3B). Especially, when infiltration was low during treatment, *PHOX2B* remained positive, while other ADRN markers were not expressed (Supplemental Fig. 3C), highlighting high sensitivity of *PHOX2B* in MRD detection.

We also observed heterogeneity of GD2 and CD56 expression in 5/108 (5%) investigated patients. These presented with notably heterogeneous levels of GD2 and/or CD56, or their complete absence, either at diagnosis or after marker expression was lost upon treatment or relapse. This is exemplified by patient 166, who initially presented with > 90% tumor cells in the bone marrow and high levels of GD2 and CD56. This patient displayed slow molecular response to induction chemotherapy and reached MRD-negative status after the consolidation phase, which was followed by anti-GD2 immunotherapy (dinutuximab beta). At relapse, bone marrow DTCs reached 1.59%, but tumor cells were negative for GD2 and the fraction of CD56-expressing cells was lower, while all tumor cells carried the neuroblastoma-typical *MYCN* oncogene amplification (Fig. 3B & E). Heterogeneity in tumor marker and immunotherapy target expression was further investigated using a more extensive multiplex imaging approach (imaging mass cytometry). This revealed, at diagnosis, next to high levels of GD2 and CD56, high expression of adrenergic neuroblast markers GATA-3, PRPH, ELAVL4 and CD24, intermediate expression of the chromaffin marker CHGA, and bone marrow homing marker CXCR4 and Ki-67, indicating proliferative activity. Only a small fraction of cells carried neural progenitor-specific SOX10 and S100B, or MES markers CD44 and vimentin. In contrast, at relapse, the phenotype of tumor cells shifted drastically, with only CD56, GATA-3 and ELAVL4 expression being retained, albeit with high variance at the single-cell level (Fig. 3F & G). Together, these data show that AIPF can identify patient tumor cells with heterogeneous expression or absence of immunotherapy targets GD2 and CD56, which might be associated with insufficient response to immunotherapy and relapse. Thus, AIPF and RT-qPCR are complementary approaches to detect MRD with high sensitivity and characterize tumor cell phenotypes as well as therapeutic targets.

### Mesenchymal markers identify MRD in adrenergic-negative and AIPF-negative bone marrow liquid biopsies prior to relapse

**Fig. 4:**
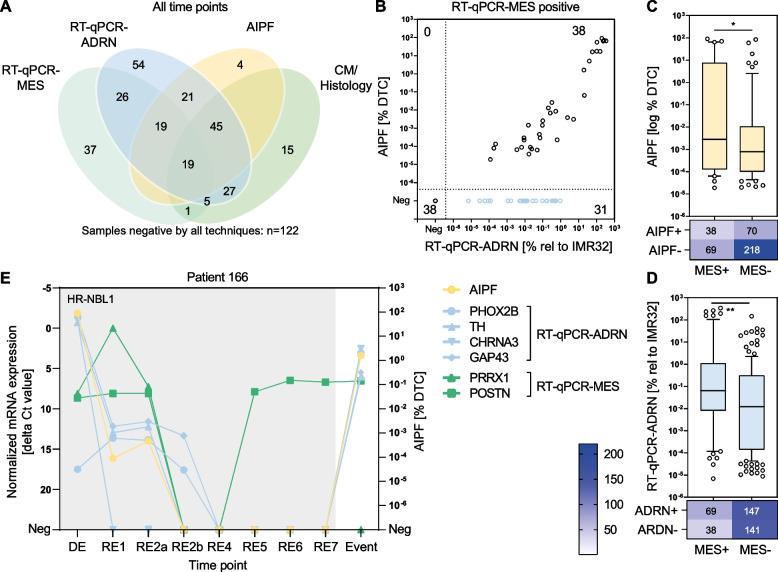
Mesenchymal markers identify MRD in adrenergic negative and AIPF negative bone marrow liquid biopsies prior to relapse. **A** Contribution of mesenchymal mRNA RT-qPCR-markers (RT-qPCR-MES),RT-qPCR-ADRN, AIPF and cytomorphology (CM)/histology. Venn diagram shows samples positive for at least one technology. Each circle represents positive results of one technique. *N* = 395 samples analyzed by all techniques; *N* = 273 samples positive by ≥ 1 technique. **B** MES-positive samples by RT-qPCR-MES. Level of infiltration according to AIPF (y-axis; given as % DTCs) and RT-qPCR-ARDN (x-axis; given as % relative to neuroblastoma cell lines IMR32) (*n* = 107; Spearman correlation = 0.59, 95% CI 0.33–0.77; *p* < 0.001 of samples positive for both techniques (*n* = 38). **C** Level of infiltration by AIPF (y-axis; given as % DTCs) versus MES-positivity (x-axis). Box plots represent 10–90 percentiles, line shows median. Heatmap represents number of negative and positive cases. * = 0.0377. **D** Level of infiltration by RT-qPCR-ADRN (y-axis; given as % relative to neuroblastoma cell line IMR32) versus MES-positivity (x-axis). Box plots represent 10–90 percentiles, line shows median. Heatmap represents number of negative and positive cases. ** = 0.0029. **E** Representative case (patient 166) showing MES marker positivity prior to relapse. At diagnosis adrenal tumor with *MYCN* amplification and multiple bone metastases and bone marrow infiltration; patient suffered a relapse one month after completion of SIOPEN/HR-NBL1 treatment and died 20 months later from disease. Bone marrow samples assessed by RT-qPCR-ADRN and RT-qPCR-MES (normalized mRNA expression left y-axis) and AIPF (given as % DTCs, right y-axis) per timepoint (x-axis). DE = diagnosis, RE1 = mid-induction chemotherapy, RE2 = end of induction therapy, RE3 = surgery, RE4 = before stem cell transplantation, RE5 = before immunotherapy, RES6 = mid-immunotherapy, RE7 = at end of immunotherapy

Recent reports suggest that neuroblastoma cells can acquire a MES phenotype in vitro and *in vivo* [20, 21]. As ADRN markers do not detect MES cells and there is reduced expression of GD2 [20, 25], we investigated *POSTN* and *PRRX1* mRNA expression as markers for MES cells. All 395 bone marrow samples were investigated by RT-qPCR-ADRN, RT-qPCR-MES, AIPF and the standard-of-care CM or histology. Of these, 273 (69%) were positive by at least one of the techniques (Supplemental Fig. 4A). Thirty-nine percent (107/273) were positive for RT-qPCR-MES and 37/273 (14%) showed exclusive positivity for MES mRNAs and were negative by the other techniques (Fig. 4A). Interestingly, when focusing only on the diagnosis timepoint, none of the samples tested positive by RT-qPCR-MES alone (Supplemental Fig. 4B). When combining AIPF with both RT-qPCR methods, 258/395 cases (65%) were RT-qPCR-ADRN^pos^MES^pos^AIPF^pos^; 161 (62%) of these were CM/histology^neg^. A total of 112/395 cases (28%) were CM/histology^pos^, and fifteen (13%) of these were RT-qPCR-ADRN^neg^MES^neg^AIPF^neg^. Accordingly, the combination of RT-qPCR-ADRN, -MES and AIPF detected a significantly higher number of samples as positive (*p* < 0.0001, Mc Nemar’s test) (Table 3).

When considering only discordant pairs, the number of samples positive for the combination but CM/histology^neg^ was 10.7 times higher (95% CI, 6.3–18.2) than the number of samples positive by CM/histology alone (Table 3), demonstrating that the combined test is superior to the current standard-of-care, CM/histology.

Of the 37 samples exclusively positive for MES mRNAs and negative by the other techniques, fourteen cases were from Dutch patients for which 12/14 plasma samples were available. In all twelve samples we detected hypermethylated *RASSF1A* DNA, a marker for neuroblastoma for which our group previously designed a droplet digital PCR assay [53].

We further investigated eight samples from patients carrying a *MYCN* amplification in tumor cells which were RT-qPCR-MES^pos^AIPF^neg^ (five RT-qPCR-ADRN^neg^, three -ADRN^pos^); however, using *MYCN* iFISH, no cells with *MYCN* amplification were detectable. For 38 cases positive for AIPF, RT-qPCR-MES and -ADRN, tumor cell infiltration covered a range of 1 DTC in 5.30 million to 2.23 million DTCs in 2.48 million MNCs according to AIPF and 0.0001–341% (relative to IMR32) according to RT-qPCR, and correlated well (Fig. 4B). There was a slightly higher tumor cell infiltration detectable in bone marrow-positive compared to those negative for MES mRNAs, as determined by AIPF (the number of investigated MNCs did not differ between MES^pos^ and MES^neg^) and RT-qPCR-ADRN (Fig. 4C & D and Supplemental Fig. 4C). This indicates that RT-qPCR-MES shows lower sensitivity, yet might identify MRD in an additional set of samples. Interestingly, in patients with serially collected samples along multi-modal treatment and follow-up, signals often alternated between different techniques. This is exemplified by patient 166 and others, who showed an initial increase of MES mRNAs and delayed clearance upon induction chemotherapy, with an early increase of MES mRNA *POSTN* prior to relapse (Fig. 4E and Supplemental Fig. 5A-C**)**.

## Discussion

Sensitive bone marrow MRD detection is crucial for evaluating therapy response, monitoring disease progression, and early relapse detection, helping to identify patients who may benefit from alternative treatments. Additionally, assessing tumor cell state plasticity and the abundance of immunotherapy targets is essential for accurately evaluating immunotherapy efficacy. In this prospective study, we address these needs using multi-modal, high-sensitivity MRD techniques in 509 samples obtained from 108 patients. We show that (1) performing all three high-sensitivity analyses, i.e. AIPF, RT-qPCR-ADRN and -MES on the same bone marrow specimen, alongside standard-of-care assessments, is feasible in an international multicenter setting; (2) multi-modal analysis is more effective at detecting bone marrow MRD than the current standard of care, with low tumor cell burden detected by RT-qPCR-ADRN more frequently and less frequently by AIPF; (3) bone marrow disease is present on both sides when DTC infiltration is high (e.g., at diagnosis). In MRD settings during and post-treatment, more frequently only one side is affected; (4) AIPF and RT-qPCR are complementary approaches for high-sensitivity MRD detection and characterizing of tumor cell phenotypes and therapeutic targets; and (5) RT-qPCR-MES shows lower sensitivity but may identify MRD in an additional subset of samples. Notably, in a patient with initially high MES markers, these markers showed delayed clearance post-induction chemotherapy and an early increase before relapse.

We have previously demonstrated the superior sensitivity of mRNA- and DTC-based bone marrow MRD detection techniques. RT-qPCR-based testing of bone marrow and peripheral blood neuroblastoma patients is a robust and highly sensitive method for detecting residual disease [19, 31–34, 36–40, 54, 55]. Stutterheim, et al*.* reported RT-qPCR positivity in 31% (5/16) of bone marrow GD2-immunocytology^neg^ samples [47] and Hartomo, et al*.* reported RT-qPCR positivity in 48% (30/63) of bone marrow-cytology^neg^ samples [56]. We have further shown in 345 patients that molecular detection of MRD by RT-qPCR was more sensitive and had higher prognostic value than GD2-immunocytology for neuroblastoma BM infiltration [57]. In contrast, AIPF can detect up to one tumor cell per million cells and provides a more automated and objective alternative to manual CM or immunocytology assessment based solely on GD2 [30]. AIPF has been compared against flow cytometry and cytomorphology in a study by Schriegel, et al. involving 369 bone marrow aspirates [4]. In the latter study, AIPF was demonstrated to be the single most sensitive method for the detection of bone marrow involvement in neuroblastoma [4]. Furthermore, at end of induction chemotherapy, DTC-positivity by AIPF was significantly associated with event-free survival and cumulative incidence of relapse in a study of 180 patients (personal communication).

In the prospective study described here, we combined these complementary techniques, i.e. RT-qPCR-ADRN, AIPF and RT-qPCR-MES, to evaluate their effectiveness in detecting MRD across 509 bone marrow aspirates from 108 patients in a multicenter setting. Our findings indicate that multicenter exchange is feasible within a clinically relevant six-day turnaround time. Despite differences in treatment protocols (NCT01704716, NCT01704716) and cut-offs for CM assessment [18], and differences in mean cell counts (3.80 million (range 130,000–7.97 million) for Austrian and 1.95 million (range 237,000–5.00 million) for Dutch samples) no major differences in results were observed between Austrian and Dutch samples compared to aggregated results. This underlines the ability to perform high-sensitivity analyses from small bone marrow volumes in an international setting and demonstrates the robustness of the techniques, paving the way for standardized MRD detection across clinical settings. This standardization is important for the ongoing European SIOPEN High-Risk Neuroblastoma 2 (HR-NBL2) trial (NCT04221035), ensuring accurate and timely diagnosis. Moreover, in our study, RT-qPCR-ADRN emerges as the most sensitive method for MRD detection. It is important to emphasize that AIPF uniquely identified certain samples not detected by RT-qPCR, suggesting that AIPF provides complementary information by enabling direct visualization and genetic verification of disseminated tumor cells, thereby offering insights into tumor cell morphology and immunotherapy marker expression that molecular methods alone cannot provide. As AIPF quantifies actual cells while RT-qPCR measures transcripts, a strong correlation between the techniques is evident when tumor cell infiltration is high, i.e., at diagnosis and at an event. Our findings confirm previous observations that *PHOX2B* sensitivity is most evident in samples with low tumor infiltration, such as bone marrow samples during treatment [41]. Notably, bone marrow infiltration levels detected by AIPF and RT-qPCR decreased during treatment and increased at relapse, reinforcing the potential of these methods response monitoring and early relapse, as well as highlighting the importance of serial sampling. Future studies should explore their association in treatment response as predictive value for survival. While our study did not recommend a reduction of bilateral BM sampling, the sensitivity of AIPF and RT-qPCR suggests unilateral sampling at diagnosis may suffice for initial bone marrow disease assessment. This could reduce patient burden and costs, with bilateral sampling reserved for follow-up when tumor cell infiltration is lower. Future studies should explore this approach. Our results also facilitate implementation as clinical decision-making tools for detecting molecular relapse, crucial for timely intervention. Prospective trials are currently underway to validate the use of RT-qPCR-ADRN as a diagnostic tool in blood liquid biopsies to determine imminent molecular relapse early. Beyond MRD detection, understanding therapy suitability is essential. Recent studies highlight distinct vulnerabilities of MES and ADRN neuroblastoma phenotypes. MES cells exhibit in vitro, more resistant to chemotherapeutics, retinoic acid, and ALK inhibition [21, 22, 58, 59], drugs that are applied in current first- and second-line treatment protocols for patients with neuroblastoma [21, 22, 58, 59]. In contrast, NOTCH pathway downregulation adversely affects MES cell survival in vitro and *in vivo* [60]. Our study shows that MES and ADRN phenotypes fluctuate during therapy, with MES-related mRNAs increasing before relapse in a patient with metastatic disease and slow induction therapy response. This corroborates previous work by our group in a retrospective cohort (95 bone marrow samples from 38 patients), where MES mRNA-expression increased in 18/27 patients during induction, while ADRN mRNA-expression initially decreased but was elevated at relapse in 9/12 patients [20]. Interestingly, MES-related mRNAs were detected in the absence of detectable ADRN mRNAs or DTCs in patients who did not relapse. Future research should determine whether these findings indicate MES tumor cells or other mesenchymal-like processes are triggered or induced by therapy or fibrosis which can result in tumor cell senescence [61, 62]. Monitoring the appearance of MES cells can provide insights into treatment response and potential resistance. Tools that accurately detect and characterize these phenotypes are useful for tailoring treatment plans to individual patients. Therapies targeting MES-neuroblastoma may benefit from such monitoring, although wider prospective studies are needed to validate these findings.

Anti-GD2 immunotherapy is a critical treatment for high-risk neuroblastoma and other cancers [63]. Our study shows that GD2 and other immunotherapy targets can be monitored and quantified using AIPF and advanced image-based multiplexed approaches. Anti-GD2 therapy, after successful trials in relapsed/refractory patients [64], is transitioning to frontline treatment as part of chemo-immunotherapy. Flow cytometry has been successfully used in 41 bone marrow specimens from 25 patients to determine GD2 levels on DTCs at diagnosis and relapse when tumor cells are abundant [28, 65]. We demonstrate that GD2 can be detected even when tumor cells are extremely rare, e.g. during treatment. Measuring GD2 and other immunotherapy targets (e.g., NCAM, L1CAM, GPC-2) in therapy-resistant cells is essential for assessing treatment efficacy and making informed clinical decisions. AIPF, as one of the few ISO-certified tests for GD2 detection, will be integral in multi-modal diagnostic workflows as antibody and CAR-T cell-based immunotherapies gain traction in neuroblastoma [25, 26, 28, 66–69] and other cancers, such as Ewing sarcoma and melanoma [70, 71], highlighting the broader applicability of these monitoring techniques.

Limitations of this study include the following aspects: First, this study is well-powered for technical comparison and proof-of-principle demonstration, however, clinical relevance of the combined test in detecting treatment response, early relapse, or evaluating its predictive or prognostic power cannot be determined. Its clinical relevance is currently being assessed prospectively in the SIOPEN frontline trial HR-NBL2 (NCT04221035) and other trials enrolling patients with high-risk neuroblastoma. A second limitation is the current clinical practice for collecting bone marrow aspirates, which mandates using the first drop of aspirates for smear preparation to assess cytomorphology [72]. Consequently, bone marrow samples used for RT-qPCR and AIPF are more diluted with blood, potentially explaining why sixteen samples were positive by CM/histology only. Lastly, AIPF is highly robust in detecting GD2 and CD56 on tumor cells, particularly when combined with iFISH, which includes adequate positive and negative controls in each analysis run. However, detecting tumor cells negative for both markers remains challenging and will require techniques with carefully designed, highly multiplexed marker panels, such as imaging mass cytometry or flow cytometry. For clinical application it will be important to carefully design such marker panels and develop in future prospective studies cost-effective lab tests for highly sensitive methods such as RT-qPCR, AIPF, and multiparameter flow cytometry.

## Conclusions

In conclusion, our study highlights the importance of advanced multi-modal MRD detection techniques, monitoring tumor phenotype switches, and assessing immunotherapy targets to improve patient outcomes in neuroblastoma and other cancers.

## Supplementary Information

Supplementary Material 1. Supplemental Figure 1.

Supplementary Material 2. Supplemental Figure 2.

Supplementary Material 3. Supplemental Figure 3.

Supplementary Material 4. Supplemental Figure 4.

Supplementary Material 5. Supplemental Figure 5.

Supplementary Material 6.

Supplementary Material 7.

Supplementary Material 8. Supplemental Data.

Supplementary Material 9. Supplemental Tables.

## Data Availability

Data used to generate the presented figures are published with this paper. Raw data will be made available upon request.

## Abbreviations

AIPF: Automatic immunofluorescence plus interphase fluorescence in situ hybridization
ADRN: Adrenergic
cDNA: Complementary DNA
CHRNA3: Cholinergic receptor nicotinic alpha 3
CI: Confidence interval
CM: Cytomorphological
DTCs: Disseminated tumor cells
EDTA: Ethylenediaminetetraacetic acid
FMO3: Flavin-containing monooxygenase 3
GAP43: Growth-associated protein 43
GD2: Disialoganglioside 2
GUSB: Glucuronidase beta
iFISH: Interphase fluorescence in situ hybridization
INSS: International Neuroblastoma Staging System
MES: Mesenchymal
MNCs: Mononuclear cells
MRD: Minimal residual disease
NCAM: Neural cell adhesion molecule
PHOX2B: Paired-like homeobox 2b
POSTN: Periostin
PRRX1: Paired related homeobox 1
RT-qPCR: Reverse transcriptase quantitative polymerase chain reaction
TH: Tyrosine hydroxylase
