# Supplementary material for: Sensitive detection of minimal residual disease and immunotherapy targets by multi-modal bone marrow analysis in high-risk neuroblastoma – a multi-center study
Source: J Exp Clin Cancer Res. 2025 Aug 2;44:224. doi: 10.1186/s13046-025-03481-w (PMC12317575; doi:10.1186/s13046-025-03481-w)
Supplement: Supplementary file 1 — Supplementary Material 1. Supplemental Figure 1. [file 13046_2025_3481_MOESM1_ESM.pdf]

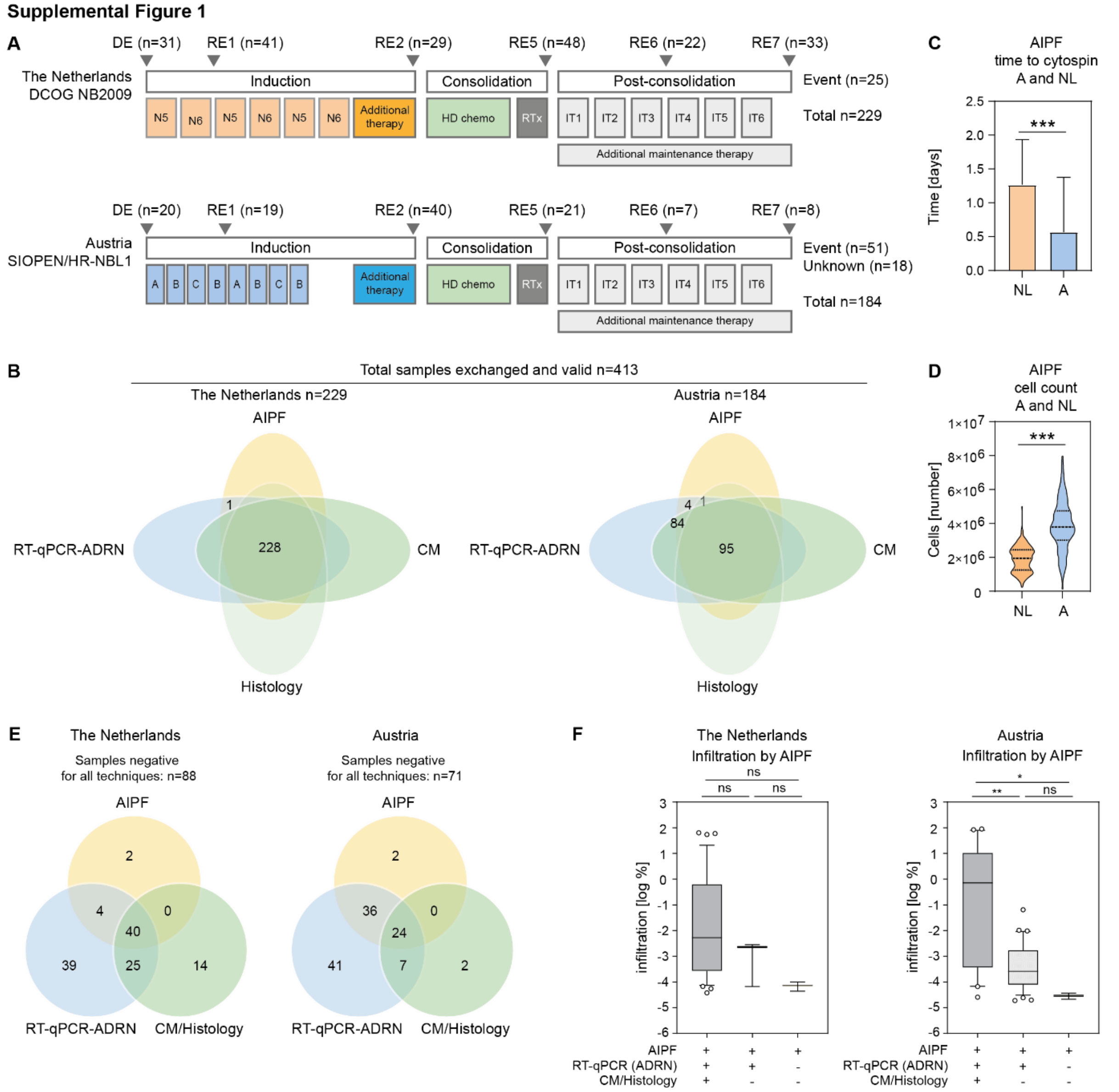


**Supplemental Figure 1. Sample collection, characteristics and analyses performed.**

(A) Schematic overview of Dutch (DCOG NBL2009[44]) and Austrian (SIOPEN/HR-NBL1[26,43]) trials. Dutch patients received 6 alternating courses of N5- and N6-chemotherapy. Austrian patients received 8 rapidly alternating courses of cisplatin, vincristine, carboplatin, etoposide and cyclophosphamine. After induction therapy, patients were treated with high dose chemotherapy with autologous hematopoietic stem cell rescue and isotretinoin for consolidation, followed by GD2-immunotherapy. Rapid COJEC induction regimen: A= vincristine, carboplatin, etoposide; B= vincristine, cisplatin; C= vincristine, etoposide, cyclophosphamide; N5/N6 induction regimen: N5= vindesine, cisplatin, etoposide; N6= vincristine, dacarbacine, ifosfamide, doxorubicin; HD chemo= melphalan, carboplatin, etoposide (DCOG NBL2009) or busulfan and melphalan (SIOPEN/HR-NBL1) followed by autologous stem cell transplantation; RTx= radiotherapy; IT= immune therapy: ch14.18/CHO +/- IL-2. Gray arrows indicate timepoints of sample acquisition. For each timepoint, number of samples for which AIPF, RT-qPCR and CM or histology is performed is given. DE= diagnosis, RE1= after cycles of therapy , RE2= at end of induction therapy , RE5= before immunotherapy, RES6= mid-immunotherapy , RE7= at end of immunotherapy.

(B) Contribution of RT-qPCR-ADRN, AIPF, cytomorphology (CM) and histology to analyzed samples per country.

(C) AIPF key performance parameter: time to cytospin preparation per country. Bars represent mean + standard deviation. NL= The Netherlands, A= Austria. ***p< 0.0001

(D) AIPF key performance parameter: cell count per cytospin preparations analyzed (cumulative for right and left side; typically, 2 cytospin preparations per side were analyzed) per country. Violin plots: dashed lines represent mean and quartiles. NL= The Netherlands, A= Austria. ***p< 0.0001

(E) Data from Figure 1C disaggregated by country. Contribution of AIPF, RT-qPCR (adrenergic (ADRN)-mRNA markers) and cytomorphology (CM)/histology. Venn diagram shows samples positive for at least one technology. Each circle represents positive results of one technique. The Netherlands n=212; Austria n=183 samples analyzed by all three techniques (total n= 395). The Netherlands n=124; Austria n=112 samples positive by ≥1 technique (total n= 236). A comparable fraction of 46/212 (21%) of Dutch and 62/183 (34%) of Austrian samples were AIPF positive.

(F) Data from Figure 1E disaggregated by country. Level of tumor cell infiltration according to AIPF (y-axis; give n as % DTCs detected by AIPF) in specimens with single or combined positivity for AIPF, RT-qPCR and CM/histology (x-axis; + positive, - negative). Box plots represent 10-90 percentiles, line shows median. **= 0.0023; ***= 0.0003.
