# Supplementary material for: Sensitive detection of minimal residual disease and immunotherapy targets by multi-modal bone marrow analysis in high-risk neuroblastoma – a multi-center study
Source: J Exp Clin Cancer Res. 2025 Aug 2;44:224. doi: 10.1186/s13046-025-03481-w (PMC12317575; doi:10.1186/s13046-025-03481-w)
Supplement: Supplementary file 2 — Supplementary Material 2. Supplemental Figure 2. [file 13046_2025_3481_MOESM2_ESM.pdf]

Supplemental Figure 2

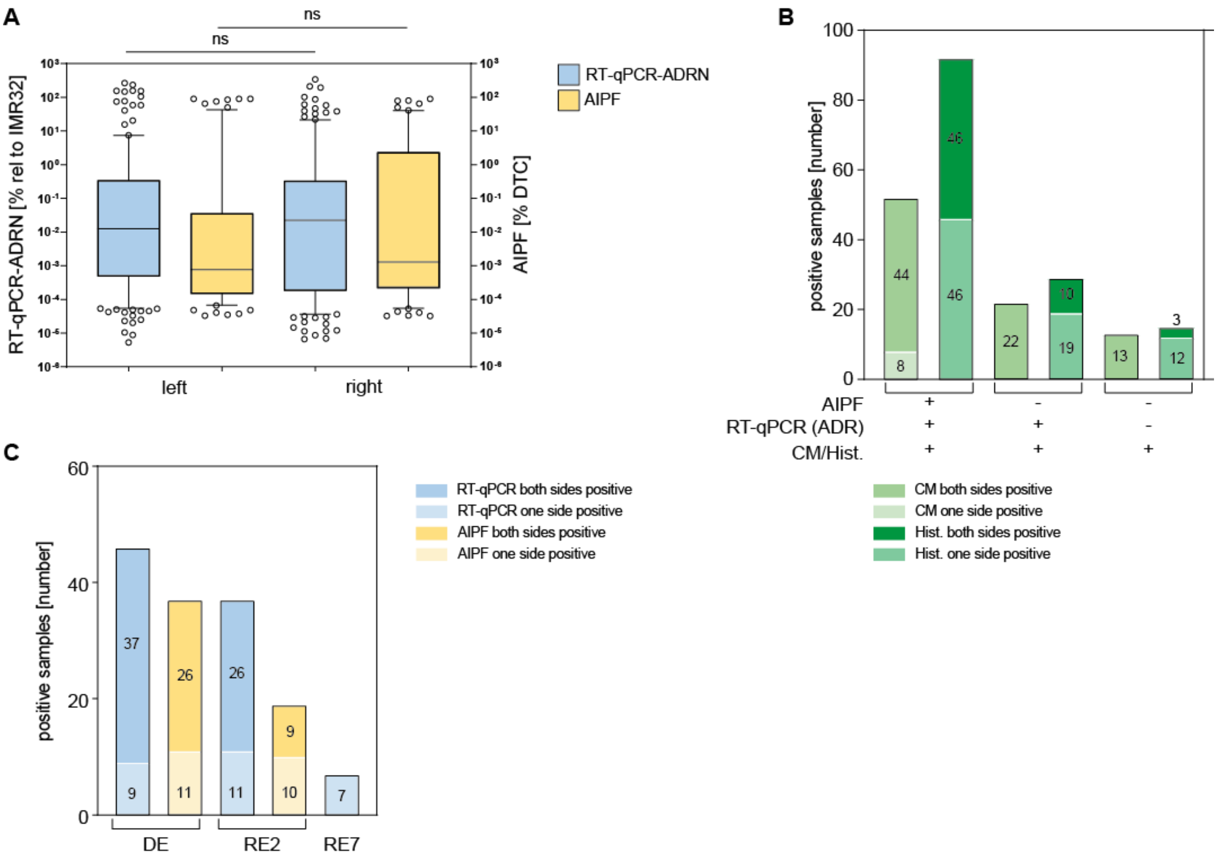

Supplemental Figure 2. Tumor cell infiltration left versus right bone marrow site.

(A) Calculated level of infiltration according to RT-qPCR (left y-axis; given as % relative to neuroblastoma cell line IMR32) and AIPF (right y-axis; given as % DTCs as detected by AIPF) in left- and right-sided samples (x-axis). Box plots represent 10 – 90 percentiles, line shows median. ns= not significant.

(B) Cytomorphology (CM) and histology positivity on one side only (unilateral) and on both sides (bilateral) in samples where both sides were analyzed for AIPF, RT-qPCR-ADRN, CM/histology (n= 87 CM samples positive; n= 136 histology samples positive).

(C) At diagnosis (DE), end of induction chemotherapy (RE2) and end of treatment (RE7) AIPF and RT-qPCR positivity on one side only (unilateral) and on both sides (bilateral) in samples where both sides were analyzed for AIPF and RT-qPCR-ADRN (n= 46 AIPF samples; n= 84 RT-qPCR-ADRN samples).
