# Supplementary material for: Sensitive detection of minimal residual disease and immunotherapy targets by multi-modal bone marrow analysis in high-risk neuroblastoma – a multi-center study
Source: J Exp Clin Cancer Res. 2025 Aug 2;44:224. doi: 10.1186/s13046-025-03481-w (PMC12317575; doi:10.1186/s13046-025-03481-w)
Supplement: Supplementary file 3 — Supplementary Material 3. Supplemental Figure 3. [file 13046_2025_3481_MOESM3_ESM.pdf]

Supplemental Figure 3

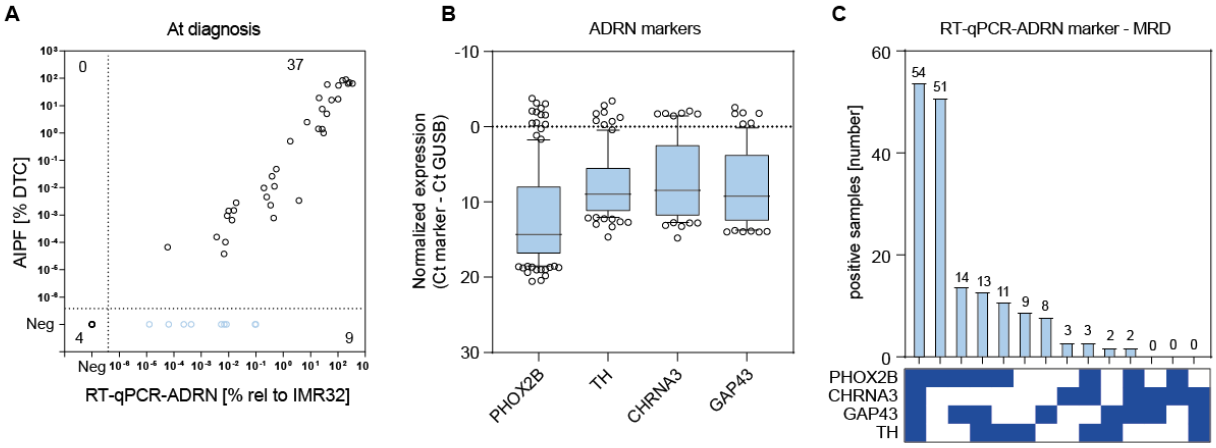

Supplemental Figure 3. Expression of adrenergic markers.

(A) Scatter plot showing level of infiltration according to AIPF (y-axis; given as % DTCs) and RT-qPCR-ADRN (x-axis; given as % relative to neuroblastoma cell line IMR32) in bone marrow samples from initial diagnosis analyzed by both techniques (n= 50; Spearman correlation= 0.75, 95% CI 0.55 -0.87; p<0.001 of samples positive for both techniques (n= 37).

(B) Normalized expression (y-axis; ΔCt value) of ADRN markers by RT-qPCR-ADRN. Box plots represent 10-90 percentiles, line shows median.

(C) Adrenergic mRNA-marker (co-)expression by RT-qPCR on samples with positive result during treatment (n= 170).
