# Supplementary material for: Sensitive detection of minimal residual disease and immunotherapy targets by multi-modal bone marrow analysis in high-risk neuroblastoma – a multi-center study
Source: J Exp Clin Cancer Res. 2025 Aug 2;44:224. doi: 10.1186/s13046-025-03481-w (PMC12317575; doi:10.1186/s13046-025-03481-w)
Supplement: Supplementary file 4 — Supplementary Material 4. Supplemental Figure 4. [file 13046_2025_3481_MOESM4_ESM.pdf]

Supplemental Figure 4

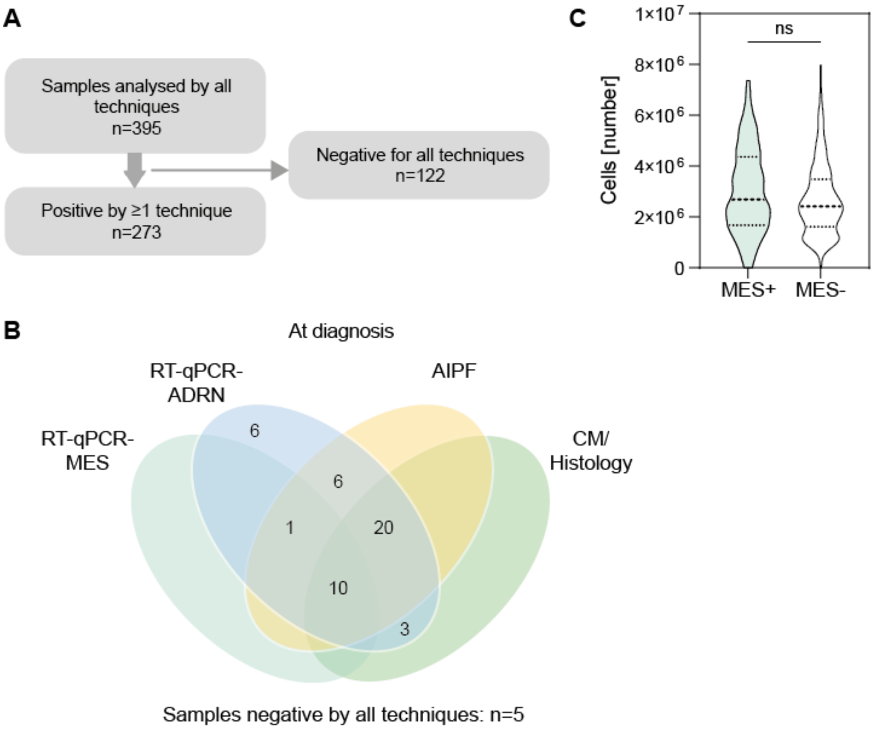

Supplemental Figure 4. Characteristics of samples positive for mesenchymal markers.

(A) Consort diagram depicting samples with multi-modal RT-qPCR-ADRN and -MES, AIPF and cytomorphology or histology analysis.

(B) Contribution of mesenchymal mRNA RT-qPCR-markers (RT-qPCR-MES), RT-qPCR-ADRN, AIPF and cytomorphology (CM)/histology at diagnosis. Venn diagram shows samples at diagnosis positive for at least one technology. Each circle represents positive results of one technique. N= 51 samples analyzed by all techniques; N= 46 samples positive by ≥1 technique.

(C) Total number of MNCs analyzed per sample in RT-qPCR-MES positive versus negative samples. Violin plots: dashed lines represent mean and quartiles. ns= not significant.
