# Supplementary material for: Sensitive detection of minimal residual disease and immunotherapy targets by multi-modal bone marrow analysis in high-risk neuroblastoma – a multi-center study
Source: J Exp Clin Cancer Res. 2025 Aug 2;44:224. doi: 10.1186/s13046-025-03481-w (PMC12317575; doi:10.1186/s13046-025-03481-w)
Supplement: Supplementary file 5 — Supplementary Material 5. Supplemental Figure 5. [file 13046_2025_3481_MOESM5_ESM.pdf]

Supplemental Figure 5

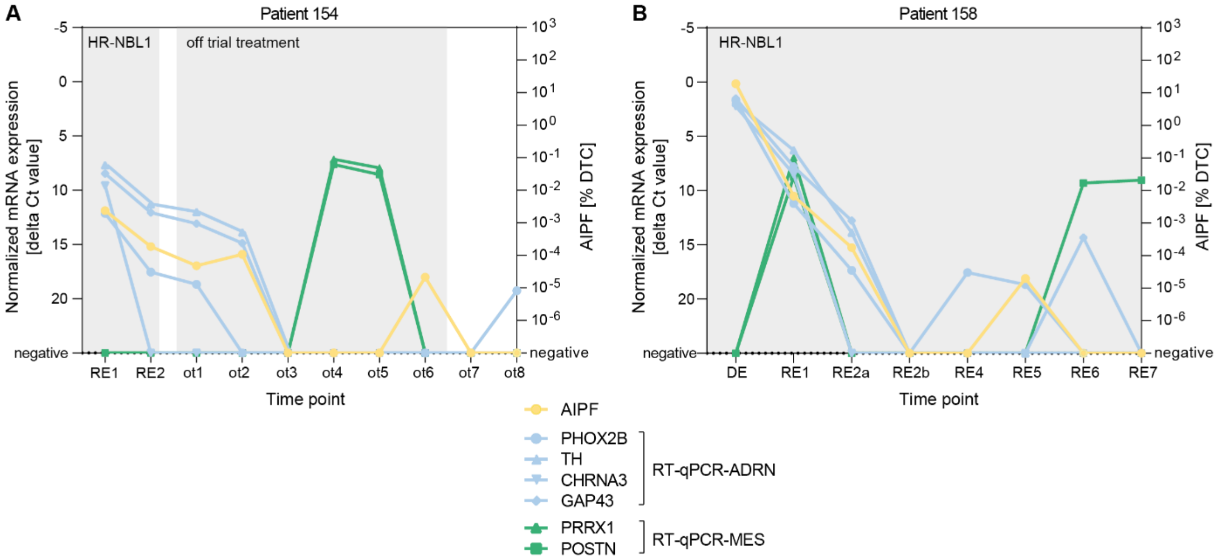

Supplemental Figure 5. Mesenchymal markers identify MRD in adrenergic negative and/or AIPF negative bone marrow liquid biopsies.

(A) – (B) Representative patient cases showing bone marrow samples assessed by RT-qPCR-ADRN and RT-qPCR-MES (left y-axis; given as normalized mRNA expression) and AIPF (right y-axis; given as % DTCs) per timepoint. DE= diagnosis, RE1= mid-induction chemotherapy, RE2= end of induction therapy, RE3= surgery, RE4= before stem cell transplantation, RE5= before immunotherapy, RES6= mid-immunotherapy, RE7= at end of immunotherapy, OT= other timepoints.

(A) Patient with non *MYCN*-amplified tumor initially treated according to SIOPEN/HR-NBL1; treatment refractory, the patient was treated off trial; partial response with residual disease; patient is alive without relapse.

(B) Patient with non *MYCN*-amplified tumor treated according to SIOPEN/HR-NBL1 was initially not responding sufficiently and received additional induction treatment. Patient is alive without relapse for >3 years.
