# Supplementary material for: Sensitive detection of minimal residual disease and immunotherapy targets by multi-modal bone marrow analysis in high-risk neuroblastoma – a multi-center study
Source: J Exp Clin Cancer Res. 2025 Aug 2;44:224. doi: 10.1186/s13046-025-03481-w (PMC12317575; doi:10.1186/s13046-025-03481-w)
Supplement: Supplementary file 8 — Supplementary Material 8. Supplemental Data. [file 13046_2025_3481_MOESM8_ESM.docx]

# Supplemental Data

**Supplemental data 1. Standard operating procedure automated immunofluorescence plus in situ fluorescence hybridization (AIPF)**

1. **Samples**

From each site 5 ml bone marrow aspirates should be collected in EDTA tubes and send within 24hrs on 4-8°C. It is highly recommended that aspirations are sampled from at least 2 different sites (usually right and left iliac crest). Sample QC is performed and sample volume and quality documented, e.g. clotting that can reduce the ability to detect DTCs, and added as remark on the report.

1. **Isolation of mononuclear bone marrow cells (MNCs) and cytospin preparation**

Here it is described how to collect the mononuclear cells (at the interface between the plasma and density gradient medium layer), wash the cells, perform erythrocyte lysis if required and prepare cytospins using e.g. a Hettich cytospin centrifuge. It is highly recommended to prepare at least 3 cytospin slides from each aspiration site containing at least 1x10^6^ to 1,2x10^6^ cells per slide (at least 3x10^6^ mononuclear cells in total). In case of low cell numbers prepare only 2 cytospin slides to reach an adequate cell density. The following steps below are also described in Swerts K et al, 2005^72^.

Material:

- 1x Phosphate Buffered Saline (PBS)

- RPMI medium

- erythrocyte lysis buffer (if required)

- density gradient medium, e.g. Ficoll / Lymphoprep

- freezing medium, e.g. Cryostor

- Hettich cytospin centrifuge, e.g. Hettich Rotofix 32, cytofunnel diameter = 20mm

- airtight slide mailing containers

- microscopy slides, e.g. Histobond

Protocol:

- overlay 10 ml bone marrow aspirate (diluted 1:1 with 1xPBS) on 7.5 mL density gradient medium

- perform density gradient centrifugation at 2100g for 25 min at room temperature without deceleration.

- collect the mononuclear cell layer and in order to wash the cells add 1xPBS, centrifuge at 250xg for 10min at 4°C and remove the supernatant up to 1cm above the cell pellet

- if required (cell pellet red) perform erythrocyte lysis: add erythrocyte lysis buffer, incubate for 10min, centrifuge at 250xg for 10min at 4°C and remove the supernatant up to 1cm above the cell pellet

- perform a second wash of the cells with 1xPBS and remove the supernatant entirely

- resuspend the cells in RPMI, count the cells and prepare cytospins using the cytospin centrifuge:

- assemble microscope slides, filter paper and funnel chamber for cytospin centrifugation
- resuspend cell suspension thoroughly by pipetting up and down
- apply 300 - 600 µL of cell suspension (~ 1,5x10^6^ cells) into the funnel chamber
- centrifuge at 1000xg for 8 min at room temperature
- aspirate supernatant and discard
- remove filter and funnel carefully, avoid contact with cell layer
- centrifuge the slides at 1300g for 4 min at room temperature

- Dry slides at room temperature for at least 1h or overnight (no fixation). Store slides in an airtight slide mailing container and store at -18-25°C. Open the container only after defrosting for at least 30min

- Preserve leftover MNCs in freezing medium in cryotubes and store at -80°C or in a liquid nitrogen tank.

1. **FITC-labelling of the anti-GD2 antibody**

Material:

- Anti-GD2 monoclonal antibody against GD2 (Disialoganglioside) received by University Tübingen CH14.18 delta CH2 4.5mg/ml.
- Borat buffer: 0,05M Borat; 0,2M NaCl; pH 9,2
- FITC (Fluorescein Isothiocyanate): Isomer I 50mg; company: Sigma N°F7250-50mg
- Dimethylsulfoxid (DMSO)
- 1% Sodium Acid solution
- 1% Bovine Serum Albumin (BSA) solution
- NAP5 columns
- 1xPBS
- rotary mixer

Protocol:

- mix 1mg FITC powder with 100ml DMSO solution and mix well; store in the dark (it has to be freshly prepared everytime, because you cannot store this solution)

- for buffering wash a NAP5 column twice with Borat buffer

- mix 250µl Anti-GD2 antibody (10mg/ml) with 250µl Borat buffer and put this solution on the column and wait until the solution runs completely through; remove the flow through

- add another 200µl Borat buffer on to the column; remove the flow through

- add 600µl of Borat buffer on to the column and collect 2x250µl buffered antibody solution

- for labelling take 250µl of the antibody solution and mix it with 5ul FITC solution

- rotate the mixture for 15min at room temperature

- in the meantime wash two new NAP5 columns three times with 1xPBS

- after rotation has finished add the FITC/antibody solution and 250µl PBS on to a new column and wait until the solution runs completely through; remove the flow through

- add 200µl 1xPBS on to the column and remove the flow through

- add 600µl 1xPBS on to the column and collect 600µl of FITC labelled antibody

- add 0,02% Sodium acid solution for preservation

- the FITC labelled anti-GD2-antibody stock solution is tested (see below) and stored at 4-8°C

For testing: prepare 1:50-1:200 dilutions with sterile filtrated BSA 2% in 1xPBS. spin down the FITC-labelled Anti-GD2 antibody stock solution to pellet the unbound fluorescence molecules before use

Test the dilutions on cytospins of GD2 positive neuroblastoma cell lines (e.g. cell line IMR32). Usually a 1:200 dilution results in good staining results.

1. **GD2/CD56 staining**

For QC a control cytospin slide with GD2-positive and negative cells (e.g. cell line IMR32 mixed with leukemic cell line HL60) must be processed with the test samples.

Material:

- Anti-GD2-FITC dilution 1:200 (see before) – freshly prepared
- CD56 Antibody dilution 1:300 with BSA 2% in 1xPBS (freshly prepared), e.g. from Ebioscience
- Mouse anti Biotin CY3 dilution 1:800 with BSA 2% in 1xPBS (freshly prepared, same procedure as for FITC-labelled Anti-GD2 antibody solution), e.g. from Dianova
- DAPI staining solution
- 2% BSA solution in 1xPBS
- 4% Formaldehyde solution
- mounting medium without DAPI staining solution, e.g. from Vectashield
- 1xPBS
- microscopy slides coverslips
- filter paper
- Staining cuvettes
- Wet chamber
- incubator

Protocol:

- Pipette 200µl 4% Formaldehyde solution on to every cytospin slide and the control slide and cover each with a coverslip

- Put the slides in a wet chamber and incubate over night at 4-8°C

- On the next day, remove the coverslip carefully and wash the slide in cuvettes of fresh 1xPBS (2x5min)

- take out the slides one at a time, shake of the 1xPBS and put it in a pre-warmed (37°C) wet chamber

- add 100µl of CD56 antibody solution 1:300 on to each slide, make sure the antibody covers the entire area of cells on the cytospin slide

- incubate all slides in the closed wet chamber at 37°C for 30min in an incubator

- after the incubation take out one slide at a time, shake off the antibody solution and store the slides in a cuvette with 1xPBS

- wash all slides with 1xPBS 2x for 5min each

- take slides out one at a time, shake off the 1xPBS and put them in a wet chamber

- add 100µl of the Mouse anti Biotin CY3 dilution 1:800 on to the cells on each cytospin slide

- incubate all slides in the closed wet chamber at 37°C for 30min in an incubator

- after the incubation take out one slide at a time, shake off the antibody solution and store the slides in a cuvette with 1xPBS

- wash all slides with 1xPBS 2x for 5min each

- take slides out one at a time, shake off the 1xPBS and put them in a wet chamber

- add 100µl of the Anti-GD2-FITC dilution 1:200 on to the cells on each cytospin slide

- incubate all slides in the closed wet chamber at 37°C for 30min in an incubator

- after the incubation take out one slide at a time, shake off the antibody solution and store the slides in a cuvette with 1xPBS

- wash all slides with 1xPBS 2x for 5min each

- take slides out – up to 10 slides at a time

- add 100µl DAPI staining dilution on to the cells on each cytospin slide

- incubate about 1min, shake off the staining solution and put all slides in a cuvette with 1xPBS

- wash all slides with 1xPBS 2x for 5min each

- Take out the slides one at a time and wipe off the bottom of the slide with a tissue

- add 10µl mounting medium and cover it with a coverslip

- gently press filter paper on top and bottom of the slides to remove excessive mounting medium

- store slides in the dark at 4-8°C for a short period only, scan and analyze immediately

1. **Automated microscopy on the Metasystems scanning system**

An Automated microscope (Axioplan 2, company Zeiss, TRITC, FITC and DAPI filter) and the software Metafer 4 (company Metasystems) are used to assess quality of the stained cytospin slide (see below), check the GD2 positive control (see below) and automatically screen all bone marrow MNCs for FITC (GD2) signal by using the software program RC Detect^30,48^. A report is generated showing the number of analyzed MNCs counted by the Isis (fluorescence imaging system) scanning system.

Images of FITC signals are captured with a 40x objective and displayed in the image gallery for review. The images and stained slides are independently reviewed by three trained and highly experienced staff according to the criteria listed below. For documentation and review digital images of positive cells and overview images including acquisition details must be stored.

1. **Analysis & interpretation**

Below is the decision tree for analysis and interpretation shown:


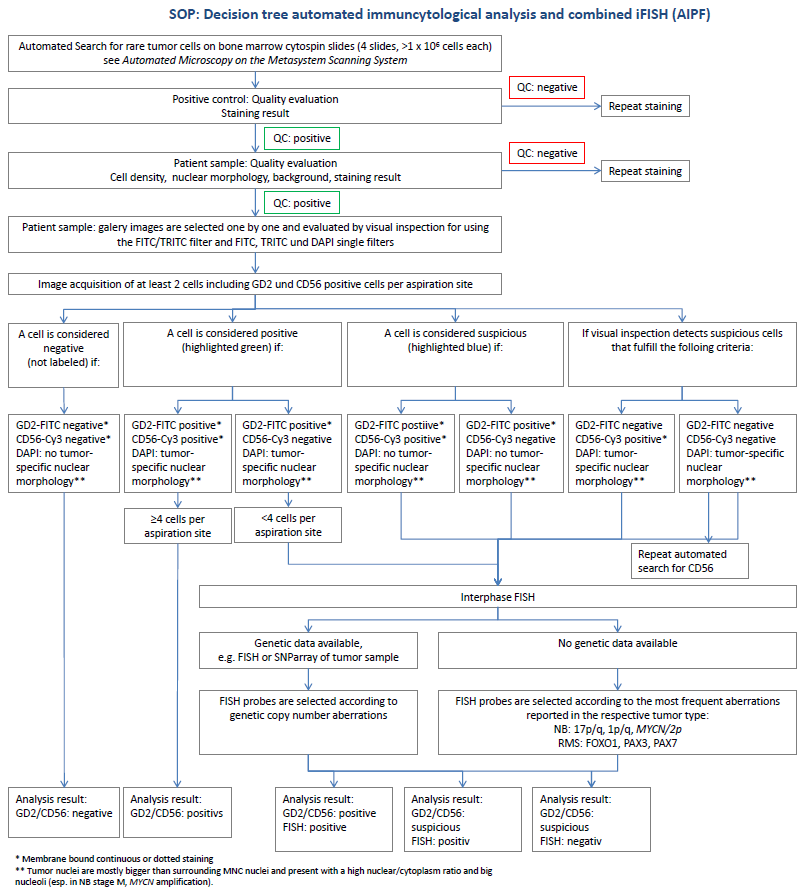


QCs

The quality of the staining on the control slide is documented electronically and on an analysis form. The control slide must show positive and specific GD2-FITC signal and low background signal onGD2 negative cells in order to proceed with analyzing the test samples.

Furthermore the quality of the stained patient’s BM aspirate cytospins is assessed by defining the following quality criteria: cell density (good, too dense with overlapping nuclei, cell density too low), cell morphology (>30% polymorphonuclear leukocytes, many smudge cells, cell swelling), GD2-FITC staining (good, weak, unspecific background signal), CD56-Cy3 staining (good, weak, unspecific background signal). If many polymorphonuclear leukocytes are seen in the sample this could be due to a contamination with blood or mobilization with GCMS and will be added on the report as a comment. Also the unspecific FITC background signal, mainly autofluorescence, will be mentioned on the report and is seen more often when there is also a low MNCs count and cell density on the stained slide. If the cell and nuclear morphology is highly abnormal this could be due to long and not adequate transport of the BM aspirate samples and therefore the result is not valid and the sample not evaluable. All QC criteria for the particular stained slide are documented on an analysis form.

MNCs count

To reach a sensitivity of 1 neuroblastoma cell in 1x10^6^ MNCs, IC must be reported on 3x10^6^ MNCs per aspirate. If the 3x10^6^ MNCs per aspirate are not reached there will be a comment on the report. If the cell count is <8x10^5^ MNCs the result is not valid and sample not evaluable.

Criteria for neuroblasts / DTCs

Cytomorphological criteria to distinguish GD2-positive neuroblasts from e.g. GD2-positive macrophages or other hematopoietic cells have been detailed previously^35^. By using the automated immunofluorescence plus FISH (AIPF) technique not only a second antibody for co-staining is used (CD56/NCAM) but also the genetic aberrations of the suspected cells can be determined by FISH [Mehes G., 2003].

For every image the GD2-FITC and CD56-Cy3 staining as well as nucleus morphology (DAPI) is considered to identify neuroblasts. The GD2-FITC and CD56-Cy3 staining is normally membrane bound and continuous or dotted (example image of staining and a tumor cell clump see below). The nucleus of neuroblasts is mostly bigger than surrounding bone marrow MNCs and with a higher nuclear/cytoplasm ratio and occasionally with nucleoli.

If there is a hint for the presence of tumor cells because of abnormal nucleus morphology but the GD2-FITC staining is negative on those cells the automated search is repeated for CD56. This rarely occurs and can be due to reduced/lost GD2 expression of the neuroblasts.


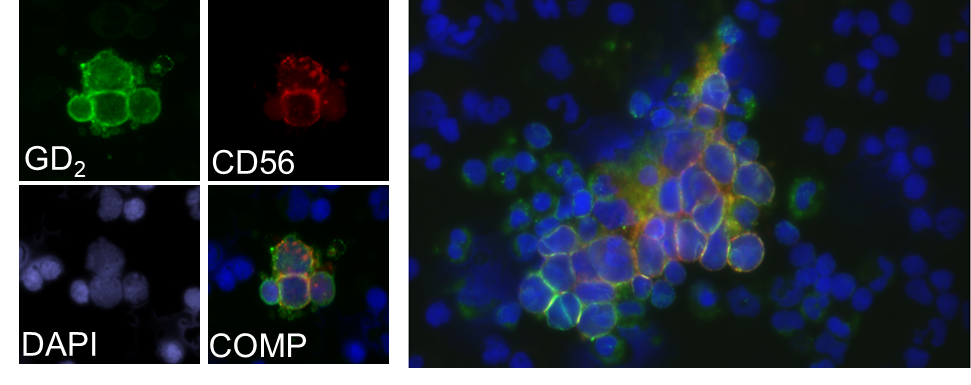


In total three examiners go through all images, applying and documenting the three criteria to identify DTCs (GD2-FITC, CD56-Cy3 and nuclear morphology) and in the end come to a final result: negative, positive or not evaluable.

**Additional Fluorescence In Situ Hybridization (FISH) analysis**

If one of the criteria’s (GD2-FITC, CD56-Cy3 and nuclear morphology) is not clearly interpretable and also if <4 cells per aspiration site are considered as neuroblasts (all three criteria positive) a FISH analysis on the same cytospin is performed in order to obtain additional genetic information of the suspected cells. After FISH preparation on the same cytospin slide the suspected cells are relocated on the automated microscope and the FISH signal pattern interpreted and documented.

To confirm the suspected cells as tumor cells we use FISH probes to detect the known aberrations (copy number changes or *MYCN* amplification) of the tumor cells. If genetic data on the primary tumor cells is not available or cannot be determined, (e.g. no tumor biopsy material) FISH probes for the most frequent aberrations in neuroblastoma are used.

1. **Reporting**

The number of neuroblasts (DTCs) and the total number of investigated cells (MNCs) per aspiration site are reported, from which the percentage of tumor infiltration can be calculated. In addition to the counts also a statement and interpretation of the result is written as a text below, including comments on the quality of the sample and possible limitations on the interpretation and conclusion.

Furthermore, it is mentioned if FISH analysis was performed to confirm suspected cells and how many cells could be identified by this.

**Supplemental data 2. Raw data and statistical analysis used to generate the figures of this study.**
