# Supplementary material for: Sensitive detection of minimal residual disease and immunotherapy targets by multi-modal bone marrow analysis in high-risk neuroblastoma – a multi-center study
Source: J Exp Clin Cancer Res. 2025 Aug 2;44:224. doi: 10.1186/s13046-025-03481-w (PMC12317575; doi:10.1186/s13046-025-03481-w)
Supplement: Supplementary file 9 — Supplementary Material 9. Supplemental Tables. [file 13046_2025_3481_MOESM9_ESM.docx]

# Supplemental Tables

**Supplemental Table 1.**

1. **List of antibodies used for Automatic immunofluorescence plus iFISH (AIPF).**

| Antibody | Species | Supplier | Conjugate | Supplier | Staining dilution |
| --- | --- | --- | --- | --- | --- |
| Anti-GD2 (Disialo-ganglioside) | ch14.18/ delta CH2 | University Tübingen, Germany | FITC (Fluorescein Isothiocyanate) | Sigma | 1:200 |
| CD56 |  | Ebioscience | Mouse anti Biotin CY3 | Dianova | 1:300 |

1. **List of antibodies used for Imaging mass cytometry (IMC).**

| Protein/ Identifier | Clone | Species | Reactivity | Supplier | Metal Tag | Staining dilution [µg/mL] |
| --- | --- | --- | --- | --- | --- | --- |
| GD2 | ch14.18/ delta CH2 | Chinese hamster/ humanized | Human | University Tübingen, Germany | Gd155 | 5 |
| CD56 | B-A19 | Mouse | Human | Immunotools | Er170 | 5 |
| CHGA | E8X7R | Rabbit | Human | CST | Dy164 | 8 |
| S100B | EP1576Y | Rabbit | Human, Mouse | Abcam | Dy161 | 5 |
| CXCR4 | REA649 | Recombinant human | Human | Miltenyi Biotec | Tb159 | 8 |
| HuC/D | 16A11 | Mouse | Human | ThermoFisher | Yb174 | 7 |
| GATA3 | L50-823 | Mouse | Human, Mouse | Becton Dickinson | Er168 | 8 |
| PRPH | polyclonal | Rabbit | Human, Mouse | ThermoFisher | Nd144 | 8 |
| SOX10 | 20B7 | Mouse | Human, Rat | Bio-Techne | Dy162 | 7 |
| VIM | EPR3776 | Rabbit | Human, Mouse | Abcam | Pt196 | 5 |
| CD44 | polyclonal | Sheep | Human | Bio-Techne | In115 | 5 |
| CD24 | ML5 | Mouse | Human | Becton Dickinson | Eu151 | 5 |
| Ki-67 | B56 | Mouse | Human, Mouse | Becton Dickinson | Tm169 | 7 |
| Iridium |  |  |  | Fluidigm/Standard Biotools | Ir191/193 | 3.84 |

**Supplemental Table 2. Characteristics of sample positive by AIPF only.**

| Sample | Timepoint | MNCs  (total) | DTCs  (total) | %  DTCs | RT-qPCR  ADRN | RT-qPCR  MES |
| --- | --- | --- | --- | --- | --- | --- |
| 1 | Progressive disease | 1.98E+06 | 2 | 1.01E-04 | Neg | Neg |
| 2 | RE5 | 2.29E+06 | 1 (only left) | 4.37E-05 | Neg | Neg |
| 3 | Off protocol | 4.70E+06 | 1 (only right) | 2.13E-05 | Neg | Neg |
| 4 | Unknown | 2.68E+06 | 1 (only right) | 3.73E-06 | Neg | Neg |

# Supplemental Figures

**
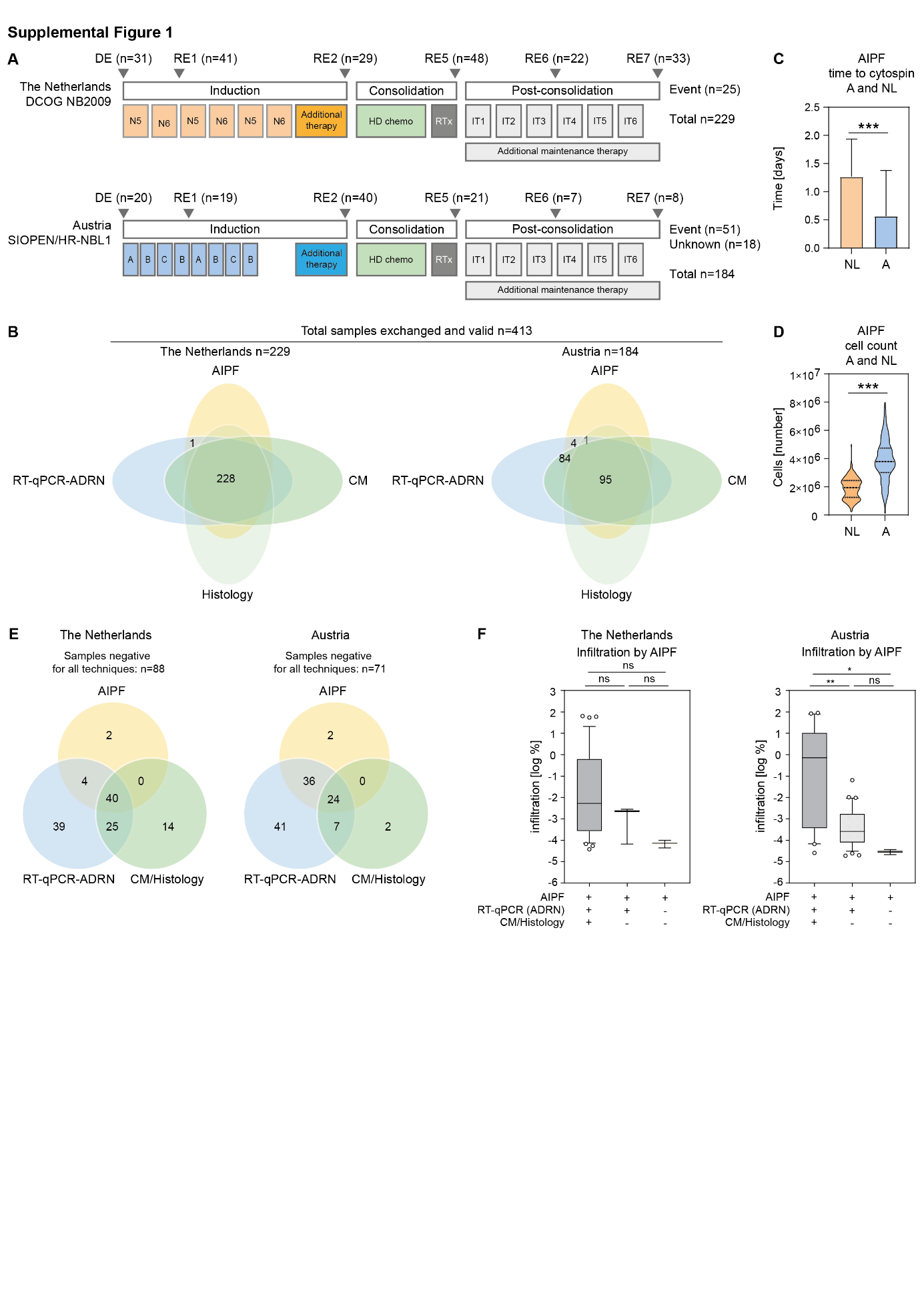
Supplemental Figure 1.** **Sample collection, characteristics and analyses performed.**

1. Schematic overview of Dutch (DCOG NBL2009^44^) and Austrian (SIOPEN/HR-NBL1^26,43^) trials. Dutch patients received 6 alternating courses of N5- and N6-chemotherapy. Austrian patients received 8 rapidly alternating courses of cisplatin, vincristine, carboplatin, etoposide and cyclophosphamine. After induction therapy, patients were treated with high dose chemotherapy with autologous hematopoietic stem cell rescue and isotretinoin for consolidation, followed by GD2-immunotherapy. Rapid COJEC induction regimen: A= vincristine, carboplatin, etoposide; B= vincristine, cisplatin; C= vincristine, etoposide, cyclophosphamide; N5/N6 induction regimen: N5= vindesine, cisplatin, etoposide; N6= vincristine, dacarbacine, ifosfamide, doxorubicin; HD chemo= melphalan, carboplatin, etoposide (DCOG NBL2009) or busulfan and melphalan (SIOPEN/HR-NBL1) followed by autologous stem cell transplantation; RTx= radiotherapy; IT= immune therapy: ch14.18/CHO +/- IL-2. Gray arrows indicate timepoints of sample acquisition. For each timepoint, number of samples for which AIPF, RT-qPCR and CM or histology is performed is given. DE= diagnosis, RE1= after cycles of therapy , RE2= at end of induction therapy , RE5= before immunotherapy, RES6= mid-immunotherapy , RE7= at end of immunotherapy.
2. Contribution of RT-qPCR-ADRN, AIPF, cytomorphology (CM) and histology to analyzed samples per country.
3. AIPF key performance parameter: time to cytospin preparation per country. Bars represent mean + standard deviation. NL= The Netherlands, A= Austria. ***p< 0.0001
4. AIPF key performance parameter: cell count per cytospin preparations analyzed (cumulative for right and left side; typically, 2 cytospin preparations per side were analyzed) per country. Violin plots: dashed lines represent mean and quartiles. NL= The Netherlands, A= Austria. ***p< 0.0001
5. Data from Figure 1C disaggregated by country. Contribution of AIPF, RT-qPCR (adrenergic (ADRN)-mRNA markers) and cytomorphology (CM)/histology. Venn diagram shows samples positive for at least one technology. Each circle represents positive results of one technique. The Netherlands n=212; Austria n=183 samples analyzed by all three techniques (total n= 395). The Netherlands n=124; Austria n=112 samples positive by ≥1 technique (total n= 236). A comparable fraction of 46/212 (21%) of Dutch and 62/183 (34%) of Austrian samples were AIPF positive.
6. Data from Figure 1E disaggregated by country. Level of tumor cell infiltration according to AIPF (y-axis; give n as % DTCs detected by AIPF) in specimens with single or combined positivity for AIPF, RT-qPCR and CM/histology (x-axis; + positive, - negative). Box plots represent 10-90 percentiles, line shows median. **= 0.0023; ***= 0.0003.

**
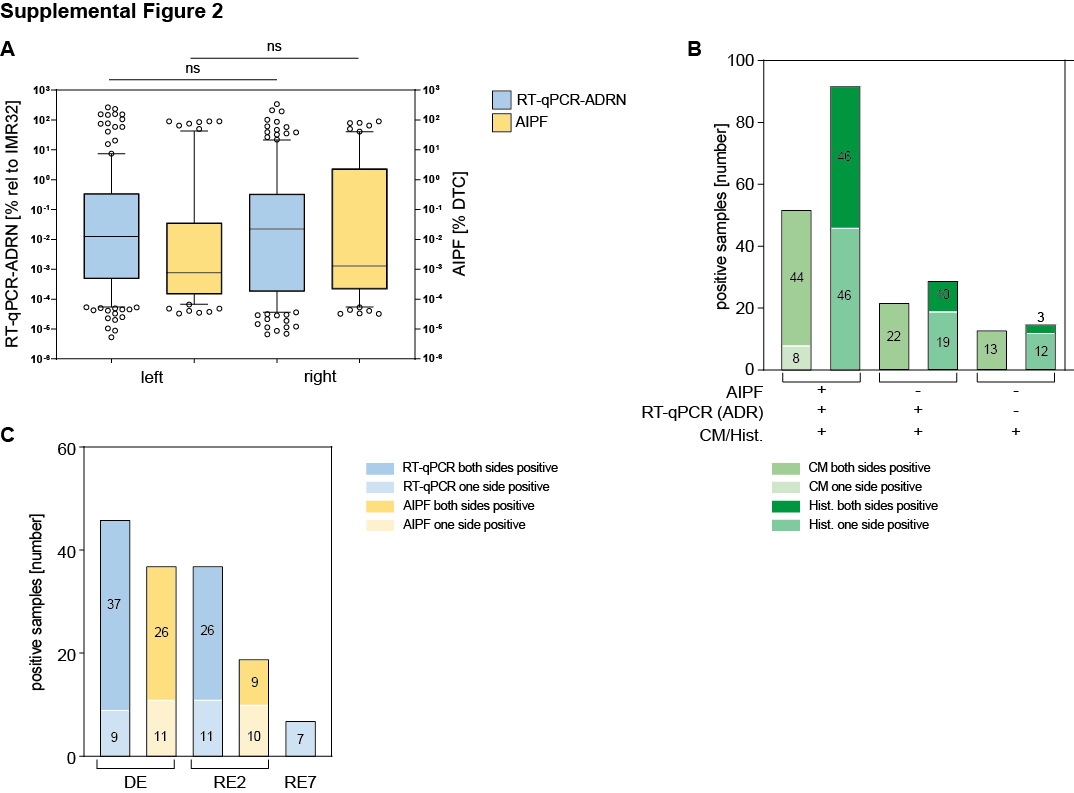
**

**Supplemental Figure 2.** **Tumor cell infiltration left versus right bone marrow site.**

1. Calculated level of infiltration according to RT-qPCR (left y-axis; given as % relative to neuroblastoma cell line IMR32) and AIPF (right y-axis; given as % DTCs as detected by AIPF) in left- and right-sided samples (x-axis). Box plots represent 10 – 90 percentiles, line shows median. ns= not significant.
2. Cytomorphology (CM) and histology positivity on one side only (unilateral) and on both sides (bilateral) in samples where both sides were analyzed for AIPF, RT-qPCR-ADRN, CM/histology (n= 87 CM samples positive; n= 136 histology samples positive).
3. At diagnosis (DE), end of induction chemotherapy (RE2) and end of treatment (RE7) AIPF and RT-qPCR positivity on one side only (unilateral) and on both sides (bilateral) in samples where both sides were analyzed for AIPF and RT-qPCR-ADRN (n= 46 AIPF samples; n= 84 RT-qPCR-ADRN samples).

**
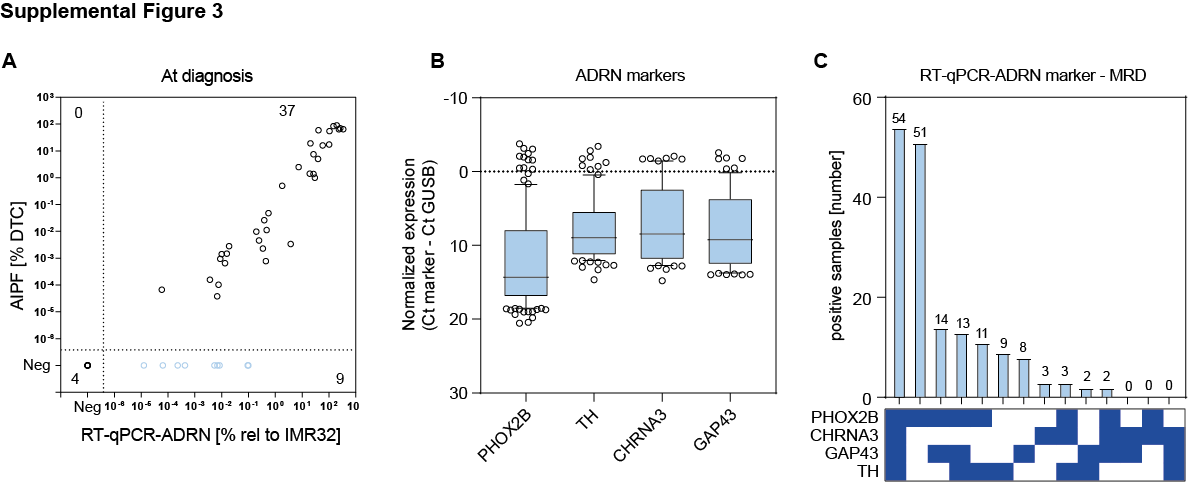
**

**Supplemental Figure 3.** **Expression of adrenergic markers.**

1. Scatter plot showing level of infiltration according to AIPF (y-axis; given as % DTCs) and RT-qPCR-ADRN (x-axis; given as % relative to neuroblastoma cell line IMR32) in bone marrow samples from initial diagnosis analyzed by both techniques (n= 50; Spearman correlation= 0.75, 95% CI 0.55-0.87; p<0.001 of samples positive for both techniques (n= 37).
2. Normalized expression (y-axis; ΔCt value) of ADRN markers by RT-qPCR-ADRN. Box plots represent 10-90 percentiles, line shows median.
3. Adrenergic mRNA-marker (co-)expression by RT-qPCR on samples with positive result during treatment (n= 170).

**
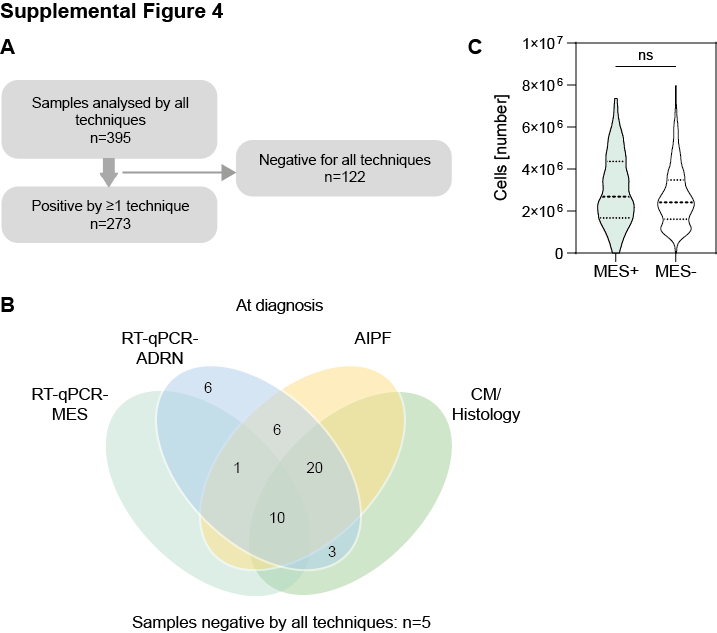
**

**Supplemental Figure 4.** **Characteristics of samples positive for mesenchymal markers.**

1. Consort diagram depicting samples with multi-modal RT-qPCR-ADRN and -MES, AIPF and cytomorphology or histology analysis.
2. Contribution of mesenchymal mRNA RT-qPCR-markers (RT-qPCR-MES), RT-qPCR-ADRN, AIPF and cytomorphology (CM)/histology at diagnosis. Venn diagram shows samples at diagnosis positive for at least one technology. Each circle represents positive results of one technique. N= 51 samples analyzed by all techniques; N= 46 samples positive by ≥1 technique.
3. Total number of MNCs analyzed per sample in RT-qPCR-MES positive versus negative samples. Violin plots: dashed lines represent mean and quartiles. ns= not significant.

**
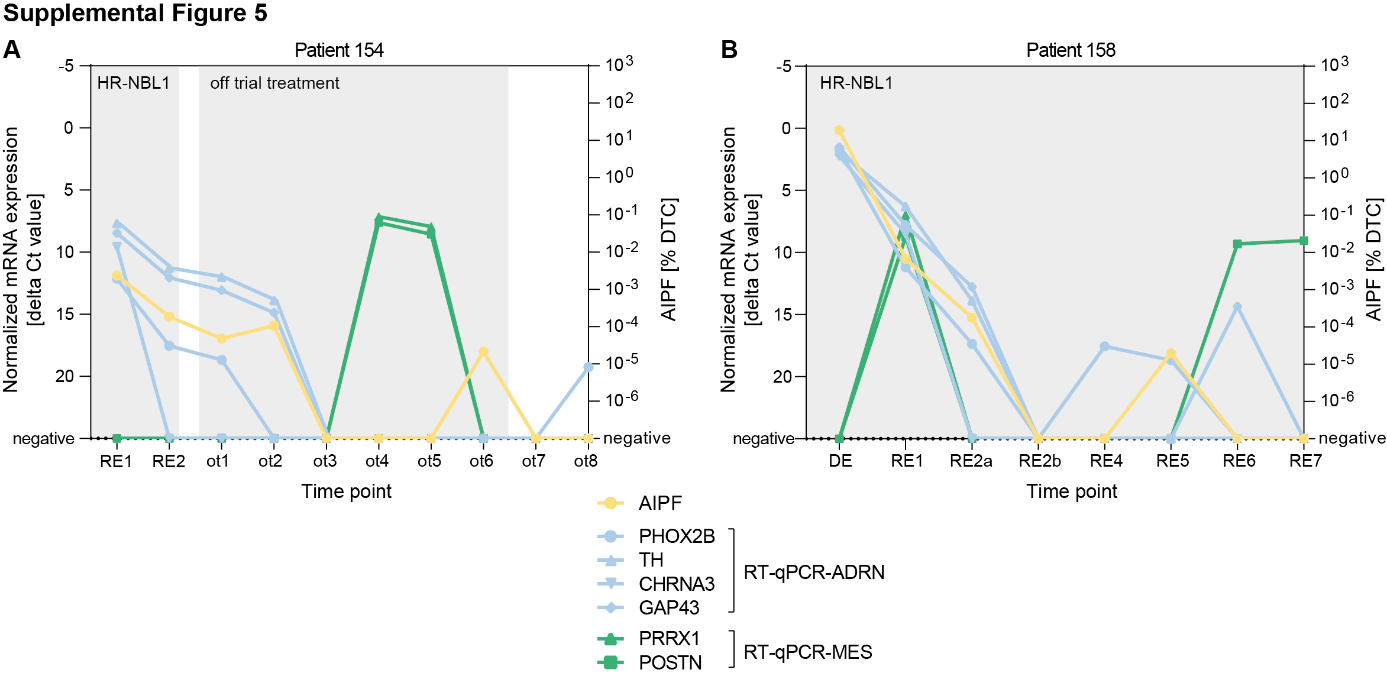
Supplemental Figure 5.** **Mesenchymal markers identify MRD in adrenergic negative and/or AIPF negative bone marrow liquid biopsies.**

1. – (B) Representative patient cases showing bone marrow samples assessed by RT-qPCR-ADRN and RT-qPCR-MES (left y-axis; given as normalized mRNA expression) and AIPF (right y-axis; given as % DTCs) per timepoint. DE= diagnosis, RE1= mid-induction chemotherapy, RE2= end of induction therapy, RE3= surgery, RE4= before stem cell transplantation, RE5= before immunotherapy, RES6= mid-immunotherapy, RE7= at end of immunotherapy, OT= other timepoints.
2. Patient with non *MYCN-*amplified tumor initially treated according to SIOPEN/HR-NBL1; treatment refractory, the patient was treated off trial; partial response with residual disease; patient is alive without relapse.
3. Patient with non *MYCN*-amplified tumor treated according to SIOPEN/HR-NBL1 was initially not responding sufficiently and received additional induction treatment. Patient is alive without relapse for >3 years.
